# Mechanistic Study of Platelet Membrane‐Coated Resveratrol Nanosystem in Mitochondrial Dysfunction and Endothelial Senescence During Atherosclerotic Lesion Development via FOXM1 Activation

**DOI:** 10.1111/acel.70632

**Published:** 2026-07-21

**Authors:** Li Xiao, Zexin Zhan, Ping Liu, Bing Qin

**Affiliations:** ^1^ Department of Neurology The Third Affiliated Hospital of Sun Yat‐Sen University Guangzhou Guangdong China; ^2^ Department of Neurology, Shandong Key Laboratory of Mitochondrial Medicine and Rare Diseases, Research Institute of Neuromuscular and Neurodegenerative Diseases Qilu Hospital of Shandong University Jinan Shandong China

**Keywords:** FOXM1, inflammatory regulation, mitochondrial membrane potential, platelet membrane‐coated nanoparticles, RNA sequencing analysis, vascular repair

## Abstract

Atherosclerosis (AS) is closely linked to endothelial cell (EC) senescence and mitochondrial dysfunction, which impair vascular repair. Resveratrol (RSV) has antioxidant, anti‐inflammatory, and pro‐angiogenic effects, but its clinical use is restricted by poor bioavailability. This study aimed to construct a platelet membrane‐coated resveratrol nanosystem (PM@RSV NPs) and investigate its mechanism of action in delaying the progression of AS by activating FOXM1 to improve mitochondrial function, inhibit EC senescence, and promote vascular regeneration. PM@RSV NPs were prepared using a solvent evaporation method combined with membrane‐coating technology, and gene expression profiles and key regulatory networks were analyzed through RNA sequencing (RNA‐seq), gene set enrichment analysis (GSEA), and least absolute shrinkage and selection operator (LASSO) regression. In vitro, PM@RSV NPs enhanced mitochondrial membrane potential and ATP generation while decreasing ROS accumulation and the number of SA‐β‐Gal‐positive cells, accompanied by FOXM1 upregulation in ECs. In vivo experiments demonstrated that PM@RSV NPs significantly reduced plaque area, improved mitochondrial function, decreased levels of senescence markers, and promoted vascular regeneration via FOXM1 regulation. In addition, PM@RSV NPs preferentially accumulated in ox‐LDL‐injured MAECs and AS lesion‐associated vascular endothelium, mainly through platelet‐membrane adhesion proteins such as GPV and P‐selectin; their biosafety was evaluated by EC viability/apoptosis assays, histological examination of major organs, and serum biochemical indices of liver and kidney function. This study confirmed that PM@RSV NPs improved mitochondrial function, inhibited endothelial senescence, and enhanced vascular regeneration by activating FOXM1, offering a novel therapeutic strategy for treating AS.

## Graphical abstract

Schematic diagram of the mechanism by which PM@RSV NPs alleviate AS via FOXM1 activation.

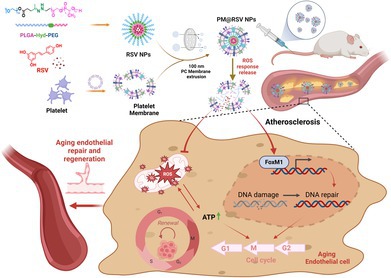

**Abbreviations**
ALT: alanine aminotransferase
ANOVA: analysis of variance
ApoE^−/^
: apolipoprotein E knockout
AS: atherosclerosis
AST: aspartate aminotransferase
ATP: adenosine triphosphate
BP: biological process
BUN: blood urea nitrogen
CC: cellular component
CLSM: confocal laser scanning microscopy
Cr: creatinine
DCM: dichloromethane
DEGs: differentially expressed genes
DLS: dynamic light scattering
ELISA: enzyme‐linked immunosorbent assay
GGPV: glycoprotein V
GO: gene ontology
GPIX: glycoprotein IX
GSEA: gene set enrichment analysis
H&E: hematoxylin and eosin
HFD: high‐fat diet
HPLC: high‐performance liquid chromatography
IHC: immunohistochemistry
IVIS: in vivo imaging system
KEGG: Kyoto encyclopedia of genes and genomes
LASSO: least absolute shrinkage and selection operator
MAECs: mouse primary aortic ECss
Mean ± SD: mean ± standard deviation
MF: molecular function
MMP: mitochondrial membrane potential
mtDNA: mitochondrial DNA
O/W: oil‐in‐water
OD: optical density
ox‐LDL: oxidized low‐density lipoprotein
PLT: platelet
PM: platelet membrane
PM@RSV NPs: platelet membrane‐coated resveratrol nanosystem
PPI: protein–protein interaction
PVA: polyvinyl alcohol
RES: reticuloendothelial system
RIN: RNA integrity number
RNA‐seq: RNA sequencing
ROS: reactive oxygen species
RSV: resveratrol
SASP: senescence‐associated secretory phenotype
SA‐β‐Gal: senescence‐associated β‐galactosidase
SPF: specific pathogen‐free
TCA: tricarboxylic acid
TEM: transmission electron microscopy

## 1 Introduction

Atherosclerosis (AS) is a chronic inflammatory vascular disorder and a major cause of cardiovascular morbidity and mortality. Its pathological features include vascular endothelium injury, lipid accumulation, inflammatory cell infiltration, and plaque formation (Roşian et al. 2025; Takahashi 2011; de Gaetano et al. 2016), ultimately resulting in vascular narrowing and restricted blood flow (Kim et al. 2024). As the disease progresses, unstable or ruptured plaques may trigger acute clinical events, such as myocardial infarction, stroke, and peripheral artery disease, posing a serious threat to human health (Momi and Gresele 2024; Saba et al. 2025). Despite the availability of lipid‐lowering agents, anti‐inflammatory drugs, and anti‐platelet (PLT) therapies, the incidence and mortality of AS remain high, especially in aging populations, where the overall disease burden continues to increase (Tariq et al. 2025). Therefore, clarifying the mechanisms underlying AS and exploring effective therapeutic strategies are of considerable clinical importance. Increasing evidence indicates that endothelial cell (EC) dysfunction and senescence are pivotal in AS progression (Gimbrone Jr. and García‐Cardeña 2016), while mitochondrial dysfunction has been identified as a key driver of EC senescence (Pulipaka et al. 2024; Yin et al. 2024). These findings offer new directions for therapeutic research in AS.

ECs are the first barrier of the vascular wall, and their functional state directly influences vascular homeostasis and disease progression (Ma et al. 2025). In AS, ECs are persistently exposed to oxidative stress, inflammatory cytokines, and metabolic disturbances, gradually entering a state of senescence (Machado‐Oliveira et al. 2020). EC senescence is marked by cell cycle arrest, impaired proliferation, DNA damage accumulation, and activation of the senescence‐associated secretory phenotype (SASP) (Savitsky et al. 2022), which further exacerbates local inflammation and vascular dysfunction (Sun et al. 2022). In addition, mitochondria, as the primary energy‐producing organelles of the cell, play a crucial role in EC senescence. Mitochondrial dysfunction results in excessive generation of reactive oxygen species (ROS) (Karnewar et al. 2022), reduced adenosine triphosphate (ATP) synthesis, and a decline in membrane potential. These alterations accelerate EC senescence, promote vascular smooth muscle cell phenotypic switching and inflammatory cell infiltration (Zha et al. 2022; Grootaert et al. 2021), ultimately contributing to plaque instability and disease progression. Thus, enhancing mitochondrial function and suppressing EC senescence may be an effective approach to delay AS progression.

Resveratrol (RSV), a naturally occurring polyphenol present in grapes, red wine, and certain traditional Chinese herbal medicines, has been widely reported to exert antioxidant, anti‐inflammatory, anti‐aging, and pro‐angiogenic effects (Yu et al. 2024), highlighting its therapeutic potential in cardiovascular diseases. For instance, RSV was found to improve mitochondrial function by activating the SIRT1 and AMPK signaling pathways (Sarkaki et al. 2021; Long et al. 2024), thereby reducing ROS production and suppressing inflammatory cytokine release. In addition, RSV promotes EC proliferation and vascular regeneration, contributing to improved vascular function (Yurdagul Jr. et al. 2014). Nevertheless, its clinical translation is hindered by low bioavailability, rapid metabolic clearance, and weak targeting efficiency. To overcome these limitations, nanodelivery systems have been developed to enhance the stability and targeting efficiency of RSV (Ahmadi et al. 2019; Ren et al. 2024). In particular, platelet membrane (PM)‐coated nanosystems have attracted considerable attention because of their excellent biocompatibility and targeting capability (Zhou et al. 2025).

FOXM1 is a pivotal transcription factor involved in cell proliferation, DNA repair, and the regulation of mitochondrial function (Black et al. 2020). Emerging evidence indicates that FOXM1 expression is strongly linked to EC proliferation and vascular regeneration (Chen et al. 2024). Specifically, FOXM1 enhances EC growth through modulation of cell‐cycle regulators, including CDK1 and Cyclin B1, while simultaneously improving mitochondrial function via activation of antioxidant‐associated genes, thereby suppressing ROS accumulation (Guan et al. 2025; van der Linden et al. 2023). In addition, FOXM1 participates in DNA damage repair and suppresses cellular senescence and apoptosis (Wang et al. 2023). In AS, FOXM1 expression is typically downregulated, which is strongly associated with EC senescence and vascular dysfunction (Bu et al. 2023). Therefore, activation of FOXM1 may serve as a novel molecular target for treating AS by improving mitochondrial function, inhibiting EC senescence, and promoting vascular regeneration. However, the specific regulatory mechanisms of FOXM1 in AS and its potential for combined therapy with RSV require further investigation.

This study developed a platelet membrane‐coated resveratrol nanosystem (PM@RSV NPs) for targeted RSV delivery and FOXM1 activation, aiming to improve mitochondrial function, suppress EC senescence, enhance vascular regeneration, and delay AS progression. These results clarify the regulatory involvement of FOXM1 in AS and provide experimental evidence supporting nanotechnology‐enabled targeted therapy. Furthermore, the favorable biosafety profile and targeting capacity of PM@RSV NPs indicate their potential for future clinical translation. Collectively, this study deepens current insight into AS pathogenesis and presents new evidence for developing innovative therapeutic strategies.

## 2 Materials and Methods

### 2.1 Ethical Statement for Animal Experiments

All animal procedures were performed in accordance with the Regulations for the Administration of Affairs Concerning Experimental Animals issued by the State Council of China (Decree No. 676) and complied with the Guide for the Care and Use of Laboratory Animals (8th edition). The experimental protocol was reviewed and approved by the Institutional Animal Care and Use Committee of Sun Yat‐Sen University (Approval No.: L102012018040Z). Mice were maintained under specific pathogen‐free (SPF) conditions at 22°C ± 2°C with 50% ± 10% relative humidity under a 12 h light/dark cycle, and were provided sterile food and water ad libitum. All procedures followed the 3Rs principle (Replacement, Reduction, and Refinement) to minimize distress. At the endpoint, mice were euthanized via intraperitoneal injection of sodium pentobarbital (100 mg/kg) according to institutional ethical guidelines.

### 2.2 Establishment of the AS Model in Apolipoprotein E Knockout (ApoE ^−/−^) Mice

Male ApoE^**−/−**^ mice aged 4–5 weeks were purchased from Beijing Vital River Laboratory Animal Technology Co. Ltd. and used to establish the AS model. Mice were fed a high‐fat diet (HFD; 21% fat, 0.15% cholesterol; D12079B, Research Diets) for 12 weeks. Age‐matched C57BL/6J mice fed a standard chow diet were used as controls for arterial lesion assessment.

The experimental grouping for PM@RSV NPs treatment was as follows:

N1. AS group: ApoE^−/−^ mice were fed an HFD for 12 weeks and administered PBS (100 μL) by tail vein injection once per week starting from Week 6, for a total of six injections.

N2. RSV‐NPs group: ApoE^−/−^ mice were fed an HFD for 12 weeks and treated with RSV‐NPs (10 mg/kg, 100 μL PBS) by tail vein injection once weekly from Week 6, for six injections.

N3. PM@RSV NPs group: ApoE^−/−^ mice were fed an HFD for 12 weeks and administered PM@RSV NPs (10 mg/kg, 100 μL PBS) via tail vein injection once weekly from Week 6, for six injections.

N4. PM@RSV NPs + sh‐NC group: ApoE^−/−^ mice received HFD feeding and PM@RSV NP treatment as described above. In addition, sh‐NC lentivirus (2 × 10^8^ TU/mouse, 100 μL PBS) was injected via the tail vein once weekly from Week 7, for three injections.

N5. PM@RSV NPs + sh‐FOXM1 group: ApoE^−/−^ mice received the same HFD feeding and PM@RSV NP treatment, together with sh‐FOXM1 lentivirus (2 × 10^8^ TU/mouse, 100 μL PBS) via tail vein injection once weekly from Week 7, for three injections (Yang et al. 2024; Zhang et al. 2025).

### 2.3 Flow Cytometric Sorting of CD31 ^+^ ECs

CD31^+^ endothelial cells (ECs) were isolated from the entire aortas (from the heart to the iliac artery) of all experimental mice, including wild type C57BL/6J mice (normal chow diet), ApoE^−^/^−^ mice (high fat diet induced AS model), and all treatment groups. All procedures were performed under sterile, RNase free, and protease free conditions to preserve the integrity of RNA, protein, and mitochondrial structure for subsequent analyses. The harvested aortas were carefully dissected to remove surrounding adipose and connective tissues, then finely minced and enzymatically digested with type I collagenase (1 mg/mL; 17,100 017, Gibco) at 37°C for 60 min. The resulting suspension was filtered through a 70 μm cell strainer (352350, Corning, USA) to obtain a homogeneous single cell suspension.

Cells were stained with Alexa Fluor 488‐conjugated anti‐CD31 antibody (ab307133, 1:100, Abcam, UK), and DAPI (1 μg/mL, #8961, Cell Signaling, USA) was used for dead cell exclusion during viability gating. CD31^+^ cells were subsequently sorted using flow cytometry (FACSAria III, BD Biosciences, USA). Post‐sort analysis confirmed a purity of over 95% and a viability exceeding 90%, as assessed by flow cytometry. These freshly sorted CD31^+^ ECs were immediately used without cryopreservation for subsequent experiments, including Western blot, MitoSOX staining, ATP measurement, oxidative stress assessment, ELISA, RT qPCR, and transmission electron microscopy. Detailed protocols for these assays are provided in Sections 2.8, 2.13 and 2.40.

### 2.4 Oil Red O Staining for Plaque Area Analysis

Mouse aortas were dissected from the heart to the iliac artery, and surrounding adipose and connective tissues were carefully removed. Specimens were fixed in 4% paraformaldehyde (PFA; P6148, Sigma‐Aldrich, USA) for 24 h and dehydrated in 30% sucrose (S0389, Sigma‐Aldrich, USA). Frozen sections (10 μm) were stained with Oil Red O (O0625, Sigma‐Aldrich, USA) at ambient temperature for 15 min, followed by three PBS washes (5 min each). Images were acquired using an Axio Observer 7 microscope (Zeiss, Germany), and plaque area ratios were quantified using ImageJ software.

### 2.5 Masson's Trichrome Staining for Fibrous Tissue Analysis

Frozen aortic sections (10 μm) were stained using a Masson's Trichrome Staining Kit (ab150669, Abcam). Following dehydration through a graded ethanol series, sections were mounted and imaged using an inverted microscope (Axio Observer 7, Zeiss, Germany). The proportion of fibrous tissue area was quantitatively analyzed using ImageJ software.

### 2.6 Histological Analysis by Hematoxylin and Eosin (H&E) Staining

Heart, liver, spleen, lung, kidney, and aortic tissues were harvested from mice. Aortic root sections containing the aortic sinuses were used for atherosclerotic lesion evaluation. Samples were fixed in 10% neutral buffered formalin (HT501128, Sigma‐Aldrich, USA) at 4°C for 24 h, followed by dehydration through graded ethanol, xylene clearing, and paraffin embedding. Paraffin sections (4 μm) were subsequently deparaffinized, rehydrated, and stained with hematoxylin (H8070, Solarbio, China) for 5 min. After differentiation in 1% acid alcohol, sections were counterstained with eosin (G1100, Solarbio, China) for 1 min. Following dehydration and mounting, tissue morphology and inflammatory infiltration in major organs were observed under a Leica DM4000B microscope (Leica Microsystems, Germany). Atherosclerotic lesions in aortic sinuses were evaluated based on luminal narrowing, foam cell accumulation, fibrous cap formation, and plaque progression.

### 2.7 Immunofluorescence Staining

For aortic tissue sections, ECs, or CD31^+^ cells isolated from mouse aortic tissues, samples were processed using appropriate fixation protocols. Specimens were fixed with 4% PFA for 15 min, washed three times with PBS, and permeabilized with 0.1% Triton X‐100 (X100, Sigma‐Aldrich, USA) for 10 min. After blocking with 5% BSA in PBS for 1 h at ambient temperature, samples were incubated overnight at 4°C with the following primary antibodies: anti‐CD31 (ab7388, 1:100, Abcam), anti‐VEGF (MA5‐32038, 1:100, Thermo Fisher), and anti‐p16 (ab51243, 1:50, Abcam). After washing with PBS, samples were incubated with fluorophore‐conjugated secondary antibodies for 1 h at ambient temperature in the dark, including Alexa Fluor 488–conjugated goat anti‐rat IgG (ab150157, 1:1000) and Alexa Fluor 647–conjugated goat anti‐rabbit IgG (ab199093, 1:1000), both from Abcam. Nuclei were counterstained with DAPI (D9542, 1:1000; Sigma‐Aldrich) for 5 min when required. Fluorescence images were captured using a fluorescence microscope (Zeiss Axio Observer 7, Germany).

### 2.8 RT‐qPCR for Gene Expression Analysis

Total RNA was extracted from ECs and CD31^+^ cells isolated from mouse aortic tissues using TRIzol reagent (15596026, Invitrogen, USA). RNA concentrations were measured with a NanoDrop spectrophotometer (Thermo Fisher, USA), ensuring that all samples had concentrations ≥ 500 ng/μL. For cDNA synthesis, 1 μg of total RNA was reverse‐transcribed using HiScript III RT SuperMix (R323‐01, Vazyme, China) under the following conditions: 42°C for 15 min and 85°C for 5 s. Quantitative PCR was carried out on a StepOnePlus system (Applied Biosystems, USA) in a 20 μL reaction mixture containing 2 μL cDNA, 0.4 μL of each primer (10 μM), and 10 μL ChamQ Universal SYBR qPCR Master Mix (Q711‐02, Vazyme, China). The PCR program included an initial denaturation step at 95°C for 5 min, followed by 40 amplification cycles of 95°C for 10 s and 60°C for 30 s. Product specificity was verified by melting curve analysis. Relative mRNA expression levels were normalized to β‐actin and calculated using the 2^−ΔΔCt^ method. Primer sequences are shown in Table S1.

### 2.9 Western Blot Analysis

Proteins were extracted from mouse ECs and CD31^+^ cells isolated from mouse aortic tissues after lysis with RIPA buffer (P0013B, Beyotime Biotechnology, China). Protein levels were quantified with a BCA assay kit (P0012S, Beyotime, China). Equal amounts of protein (20 μg/sample) were separated by SDS‐PAGE and transferred onto PVDF membranes (IPVH00010, Millipore, USA). Membranes were blocked with 5% non‐fat milk in TBST (T1085, Solarbio, China) for 1 h at ambient temperature, then incubated with primary antibodies overnight at 4°C. After washing, membranes were incubated with HRP‐conjugated anti‐mouse (#7076, 1:5000) or anti‐rabbit (#7074, 1:5000) secondary antibodies (Cell Signaling Technology, USA) for 1 h at ambient temperature. Following three TBST washes for 5 min each, protein bands were visualized using ECL reagent (Omt‐01, Omiget, China) and detected by X‐ray film exposure. Band intensities were analyzed with ImageJ, using β‐actin as the loading control. Primary antibody details are provided in Table S2.

### 2.10 MitoSOX Assay for Mitochondrial ROS

For mitochondrial ROS measurement, MAECs with culture medium removed or flow cytometry‐sorted CD31^+^ cells were maintained in serum‐free DMEM for 2 h to reduce matrix interference, followed by two washes with PBS. Mitochondrial ROS levels were assessed only in live cell samples. Cells were stained with 5 μM MitoSOX Red fluorescent probe (Mitochondrial Superoxide Indicator, S0061S, Beyotime, China) at 37°C for 30 min in the dark, followed by three washes with pre‐warmed PBS. Fluorescence signals were quantified using a flow cytometer (BD Biosciences, USA), and intracellular mitochondrial ROS levels were analyzed with FlowJo software (v10.8, Tree Star, USA).

### 2.11 ATP Production Assay

Intracellular ATP levels were measured using an ATP assay kit (S0026, Beyotime Biotechnology, China). After treatment, MAECs or flow cytometry‐sorted CD31^+^ cells were washed with pre‐cooled PBS and lysed. Cell lysates (20 μL) were mixed with 80 μL reaction solution in a 96‐well plate and quantified against an ATP standard curve. Fluorescence was measured using a Synergy H1 microplate reader (BioTek, USA) at excitation/emission wavelengths of 535/590 nm.

### 2.12 Detection of Oxidative Stress Markers (SOD, CAT, MDA)

After lysis of MAECs or CD31^+^ ECs isolated from mouse aortic tissue, antioxidant enzyme activities and lipid peroxidation levels were measured using commercial assay kits. Superoxide dismutase (SOD) activity was assessed with the Mouse Superoxide Dismutase 1 ELISA Kit (ab285309; Abcam, UK), catalase (CAT) activity with the Mouse Catalase ELISA Kit (ab222512; Abcam, UK), and malondialdehyde (MDA) levels with the Lipid Peroxidation (MDA) Assay Kit (ab118970; Abcam, UK). Absorbance was recorded at 450 or 532 nm using a SpectraMax iD5 microplate reader (Molecular Devices, USA), and results were calculated according to standard curves.

### 2.13 Enzyme‐Linked Immunosorbent Assay (ELISA)

ELISA was performed to quantify TNF‐α, IL‐6, VEGF, and Ang‐1 levels in MAECs or CD31^+^ cell homogenates from aortic tissues. Following cell lysis, samples were centrifuged at 12,000 r/min for 15 min at 4°C, and the supernatants were collected for analysis. Target protein levels were measured using the Mouse TNF‐α ELISA Kit (ab208348), IL‐6 ELISA Kit (ab222503), Mouse VEGF ELISA Kit (ab100751), and Mouse Ang‐1 ELISA Kit (ab208349), all purchased from Abcam (USA). Absorbance was read at 450 nm with a BioTek Synergy H1 multifunctional microplate reader, and cytokine concentrations were calculated accordingly.

### 2.14 Isolation and Extraction of PMs

Whole blood was collected from adult C57BL/6J mice (8 weeks, 18–20 g; *n* = 219; Beijing Vital River Laboratory Animal Technology Co. Ltd., China) and anticoagulated with 3.8% sodium citrate (C8532, Sigma‐Aldrich, USA). Blood was centrifuged at 800 × *g* for 15 min at 4°C (5810R, Eppendorf, Germany) to remove red and white blood cells. The supernatant was centrifuged at 1500 × *g* for 10 min at 4°C to pellet platelets, which were washed three times with PBS and resuspended in PBS containing protease inhibitor (11697498001, Roche, Switzerland). PMs were obtained by repeated freeze–thaw cycles (−80°C/37°C, 3–5 times). The lysate was centrifuged at 1000 × *g* for 10 min at 4°C to eliminate cellular debris, followed by ultracentrifugation of the supernatant at 100,000 × *g* for 1 h at 4°C. The resulting PM pellet was rinsed twice with PBS, resuspended in ultrapure water, and stored for subsequent use. Protein concentration was measured using a BCA assay. The integrity of membrane extraction was confirmed by WB for PLT‐specific proteins, including P‐selectin, glycoprotein V (GPV), glycoprotein IX (GPIX), and GPIIIa. The purity and identity of the PM preparation were evaluated before coating by confirming enrichment of platelet membrane markers (P‐selectin, GPV, GPIX, and GPIIIa) and by removing residual cellular debris through low‐speed centrifugation followed by ultracentrifugation; only PM preparations showing the expected platelet‐marker profile were used for nanoparticle coating.

### 2.15 Preparation of pH‐Responsive PLGA‐Hyd‐PEG@RSV Precursor Nanoparticles (Referred to as “Bare RSV NPs”)

PLGA‐Hyd‐PEG@RSV nanoparticles (hereinafter referred to as “bare RSV NPs”) were prepared using a single‐emulsion solvent evaporation method, serving as the core precursors for subsequent membrane coating. Briefly, resveratrol (RSV, R5010, Sigma‐Aldrich, USA) and PLGA‐Hyd‐PEG (R‐PL3362‐17 k, Xi'an Ruixi Biological Technology Co., China) were dissolved in dichloromethane (DCM) at a mass ratio of 1:10 to form the organic phase. The aqueous phase consisted of 1% (w/v) polyvinyl alcohol (PVA) solution. The organic phase was added dropwise to the aqueous phase and emulsified using a probe sonicator (200 W, 30 s, 5 s intervals) in an ice bath to form a stable oil‐in‐water (O/W) emulsion. The DCM was evaporated under magnetic stirring for 4 h to obtain the uncoated PLGA‐Hyd‐PEG@RSV nanoparticles (bare RSV NPs). The emulsion was centrifuged at 12,000 × *g* for 10 min at 4°C, and nanoparticles were washed three times with ultrapure water to remove unencapsulated RSV. Nanoparticles were resuspended in PBS (pH 7.4) and stored at 4°C. Particle size, zeta potential, and encapsulation efficiency were determined by dynamic light scattering (DLS) and high‐performance liquid chromatography (HPLC). pH responsiveness was assessed by comparing particle size and RSV release at pH 5.0 and pH 7.4.

### 2.16 Construction of PM@RSV NPs

To construct the platelet membrane‐coated RSV nanoplatform, bare RSV NPs were incubated with purified PMs prepared from C57BL/6J mouse platelets as described in Section 2.14 at a 1:1 mass ratio in PBS (pH 7.4) for 2 h at ambient temperature with gentle shaking to facilitate initial membrane adsorption onto the nanoparticles. Subsequently, the mixture was subjected to ultrasonication (100 W, 5 min, 5 s intervals) to facilitate membrane fusion, followed by extrusion through a 200 nm polycarbonate membrane for 10 cycles to obtain uniformly coated PM@RSV NPs. To eliminate excess free membrane vesicles, the nanoparticle suspension was centrifuged at 100,000 × *g* for 1 h at 4°C, and the collected pellets were redispersed in PBS (pH 7.4) for subsequent experiments and characterization. The particle size, zeta potential, and encapsulation efficiency of the resulting PM@RSV NPs were characterized using DLS (Zetasizer Nano ZS, Malvern Instruments, UK) and UV–visible spectrophotometry. The success of membrane coating and retention of PM purity/identity were confirmed by WB analysis of PM‐specific proteins, including P‐selectin, GPV, GPIX, and GPIIIa. pH responsiveness was assessed by monitoring particle size and RSV release at pH 5.0 and pH 7.4.

### 2.17 Measurement of Particle Size and Zeta Potential by DLS

The hydrodynamic diameter and surface charge of PM@RSV NPs were determined using DLS. Samples were dispersed in ultrapure water at 0.5 mg/mL. Each sample was measured in triplicate and averaged.

### 2.18 Evaluation of Long‐Term Stability of PM@RSV NPs

PM@RSV NPs were stored at 37°C for 24 h to assess short‐term stability and at 4°C for 14 days to evaluate long‐term stability. At designated time points, particle size and zeta potential were measured by DLS. All samples were diluted to 0.5 mg/mL, analyzed in triplicate, and averaged.

### 2.19 Transmission Electron Microscopy (TEM) Characterization

The morphology of PM@RSV NPs was examined by TEM (JEM‐1400, JEOL, Japan). A drop of the nanoparticle suspension was applied onto a carbon‐coated copper grid (01841, Ted Pella, USA) and incubated at ambient temperature for 10 min. Residual liquid was gently removed with filter paper, and the samples were negatively stained with 2% phosphotungstic acid solution (P4006, Sigma‐Aldrich, USA) for 1 min. After removal of excess staining solution and air drying, the grids were observed at an accelerating voltage of 80 kV. The uniformity and integrity of the PM coating were evaluated according to membrane thickness, coating homogeneity, and the clarity of the core–shell interface.

### 2.20 Determination of Encapsulation Efficiency and Drug Loading by HPLC

RSV encapsulation efficiency and drug loading in PM@RSV NPs were determined by HPLC (Agilent 1260 Infinity II, Agilent Technologies, USA). PM@RSV NPs were dissolved in methanol, sonicated for 5 min, and passed through a 0.22 μm membrane filter prior to analysis. Chromatographic separation was carried out on a C18 column (ZORBAX Eclipse Plus, 4.6 × 250 mm, 5 μm, Agilent Technologies, USA) using a methanol/water mobile phase (70:30, v/v) at a flow rate of 1 mL/min. Detection was performed at 306 nm with an injection volume of 20 μL. RSV standard solutions were analyzed under the same conditions to establish a standard calibration curve. RSV concentration was quantified based on the standard calibration curve. Encapsulation efficiency and drug loading were calculated as follows:

Encapsulation Efficiency (%) = (Amount of encapsulated RSV/Total RSV added) × 100.

Drug Loading (%) = (Amount of encapsulated RSV/Total weight of nanoparticles) × 100.

### 2.21 Drug Release Study

The drug release profile of PM@RSV NPs was evaluated under simulated conditions at different pH levels. Nanoparticles were dispersed in PBS buffer (pH 7.4) and acetate buffer (pH 5.0; 1.08208, Sigma‐Aldrich, USA), with the final RSV concentration adjusted to 1 mg/mL. The suspensions were transferred into dialysis bags with a molecular weight cut‐off of 10 kDa (BAF148480000, Sigma‐Aldrich) and immersed in the corresponding buffer media.

Dialysis systems were incubated at 37°C with shaking at 100 rpm to mimic in vivo conditions. At designated intervals (0.5, 1, 2, 4, 8, 12, 24, and 48 h), 20 μL of release medium was withdrawn and immediately replaced with an equal volume of fresh buffer to preserve sink conditions. RSV concentrations in the collected samples were quantified by HPLC.

### 2.22 CCK‐8 Assay for Cell Viability

Cell viability was determined using a CCK‐8 kit (C0037, Beyotime, China). MAECs were plated in 96‐well plates at 5 × 10^3^ cells/well. Upon reaching ~70% confluence, cells were treated with PM@RSV NPs (10, 25, and 50 μg/mL) for 24 h. CCK‐8 solution (10 μL) was added per well and incubated for 2 h. Absorbance at 450 nm was measured using a BioTek Synergy H1 microplate reader (USA), and relative cell viability was calculated.

### 2.23 Calcein‐AM/PI Double Staining Assay

MAECs were seeded into 6‐well plates at 1 × 10^5^ cells per well. After reaching about 70% confluence, cells were treated with PM@RSV NPs at 10, 25, or 50 μg/mL for 24 h. Cells were then rinsed with PBS and stained with a Calcein‐AM/PI viability assay kit (CA1630, Solarbio, China) at 37°C in the dark for 30 min. Fluorescence images were obtained using a Leica DMi8 microscope (Germany), where green Calcein‐AM fluorescence represented live cells and red PI fluorescence indicated dead cells. Cell viability was quantified with ImageJ software.

### 2.24 Flow Cytometry for Apoptosis Detection

MAECs were seeded in 6 cm dishes at 2 × 10^5^ cells/dish and treated with PM@RSV NPs (10, 25, and 50 μg/mL) for 24 h upon reaching 70% confluence. Cells were collected by trypsinization and centrifugation at 1000 r/min for 5 min. Apoptosis was assessed using an Annexin V‐FITC/PI detection kit (C1062S, Beyotime, China) with incubation at 4°C in the dark for 15 min. Fluorescence signals were analyzed using a BD FACSAria III flow cytometer (USA), and the proportions of early and late apoptotic cells were quantified with FlowJo software (Tree Star, USA).

### 2.25 Preparation of Cy5‐Labeled PM@RSV NPs

PM@RSV NPs were incubated with 1 mg/mL Cy5‐NHS (1C041, Lumiprobe, USA) in 0.1 M carbonate buffer (pH 8.3; C3041, Sigma‐Aldrich, USA) at ambient temperature for 2 h. Unreacted Cy5 was removed by ultrafiltration at 10,000 × *g* for 10 min using Amicon Ultra‐15 filter units (10 kDa, UFC901024, Millipore, USA). Labeled nanoparticles were resuspended in PBS for subsequent use.

### 2.26 Fluorescence Microscopy to Evaluate Targeting Ability of PM@RSV NPs

Normal and ox‐LDL‐damaged MAECs were treated with Cy5‐labeled PM@RSV NPs (50 μg/mL) for 4 h, followed by three PBS washes to remove unbound nanoparticles. Cell nuclei were counterstained with DAPI (D9542, 1:1000, Sigma‐Aldrich, USA) for 10 min at ambient temperature in the dark and rinsed three additional times with PBS. Fluorescence images were obtained using a Zeiss Axio Observer 7 microscope (Germany) under consistent exposure parameters for all samples.

### 2.27 Neutralizing Antibody Blocking Assay

Cy5‐labeled PM@RSV NPs (50 μg/mL) were preincubated with anti‐GPV antibody (PA5‐47889, Thermo Fisher) and anti‐P‐selectin antibody (ab255822, Abcam) for 30 min to block PM‐derived adhesion ligands, and the blocked nanoparticles were then incubated with ox‐LDL‐induced injured MAECs at ambient temperature for 4 h. Following three PBS washes to eliminate excess nanoparticles, the intracellular distribution of Cy5 fluorescence was visualized under a fluorescence microscope (Zeiss Axio Observer 7, Germany). The percentage of Cy5‐positive cells was quantified using flow cytometry (BD FACS Canto II, BD Biosciences, USA) to evaluate the effect of ligand blocking on nanoparticle adhesion to injured MAECs.

### 2.28 Intracellular Distribution of PM@RSV NPs

Normal MAECs and ox‐LDL‐induced injured MAECs were incubated with Cy5‐labeled PM@RSV NPs (50 μg/mL) for 4 h, then washed three times with PBS and fixed in 4% PFA for 15 min. The fixed cells were permeabilized with 0.1% Triton X‐100 (X100, Sigma‐Aldrich, USA) for 10 min, followed by immunostaining with anti‐LAMP1 antibody (ab320851, 1:500, Abcam, UK) to label early endosomes and lysosomes. Cells were then incubated with Alexa Fluor 488‐conjugated goat anti‐mouse IgG secondary antibody (ab150113, 1:1000, Abcam, UK) for 1 h. Confocal images were collected using a Zeiss LSM 880 microscope (Germany).

### 2.29 Quantification of Cellular Uptake by Flow Cytometry

Normal and ox‐LDL‐treated MAECs were incubated with Cy5‐labeled PM@RSV NPs for 1 or 4 h, followed by three washes with PBS to remove excess nanoparticles. Cy5 fluorescence intensity was subsequently analyzed using flow cytometry (BD Biosciences, USA), and nanoparticle uptake efficiency was quantified with FlowJo software.

### 2.30 RNA Sequencing (RNA‐Seq) and Data Quality Control

To further investigate the molecular mechanisms of PM@RSV NPs in AS, aortic tissues were isolated from three ApoE^−/−^ mice treated with PM@RSV NPs and three ApoE^−/−^ mice from the AS model control group. After PBS perfusion, the tissues were minced, digested, and filtered to obtain single‐cell suspensions. The cells were then stained with Alexa Fluor 488‐conjugated anti‐CD31 antibody and sorted using a flow cytometer (CytoFLEX LX, Beckman Coulter, USA) to isolate CD31^+^ ECs.

Total RNA was immediately extracted from sorted cells using TRIzol reagent (15596026, Invitrogen, USA). RNA purity (A260/A280: 1.8–2.0) and concentration were assessed using NanoDrop 2000 and Qubit 4.0 (Thermo Fisher, USA). RNA integrity was evaluated on an Agilent 2100 Bioanalyzer (Agilent Technologies, USA), requiring RIN > 7.0. Sequencing libraries were prepared and analyzed on the Illumina NovaSeq 6000 platform, generating 150 bp paired‐end reads with a sequencing depth exceeding 30 million reads per sample. Raw sequencing quality was examined using FastQC (v0.11.9), while adaptor sequences and low‐quality reads were removed with Trimmomatic (v0.39).

### 2.31 Differential Expression Analysis

Clean reads obtained after quality filtering were mapped to the mouse reference genome (GRCm38, GENCODE, https://www.gencodegenes.org/) using HISAT2 (v2.2.1). Transcript abundance was estimated with StringTie (v2.1.4) and differentially expressed genes (DEGs) were identified using DESeq2 (v1.38.0) in R with criteria of |log_2_FC| ≥ 1 and FDR < 0.05. Volcano plots were generated with the ggplot2 package (v3.4.0).

### 2.32 Gene Ontology (GO) and Kyoto Encyclopedia of Genes and Genomes (KEGG) Functional Enrichment Analysis

GO and KEGG enrichment analyses were performed on DEGs using the clusterProfiler package (v4.6.0). GO analysis covered biological process (BP), cellular component (CC), and molecular function (MF). Enriched pathways and terms with both *p*‐values and *q*‐values below 0.05 were regarded as statistically significant.

### 2.33 Gene Set Enrichment Analysis (GSEA)

GSEA was conducted using GSEA software (v4.3.2). HALLMARK and KEGG gene sets from the MSigDB (v7.5, https://www.gsea‐msigdb.org/gsea/msigdb/) were used. The analysis parameters were set as “gene_set_size = 15‐500”, and statistical significance was calculated using 1000 permutations. GSEA results were visualized using the enrichplot package (v1.18.0) in R.

### 2.34 Least Absolute Shrinkage and Selection Operator (LASSO) Regression

LASSO regression was conducted using the glmnet package in R to perform feature selection and shrinkage estimation by tuning the penalty parameter λ. LASSO was selected instead of Ridge regression because the purpose of this analysis was to identify a concise set of key genes from a high‐dimensional DEG matrix with a limited number of biological replicates. The L1 penalty used by LASSO can shrink less informative coefficients to exactly zero, thereby enabling sparse feature selection, whereas Ridge regression uses an L2 penalty that retains most variables and is more suitable for prediction or stabilizing multicollinearity than for selecting a compact gene signature (Tibshirani 1996; Hoerl and Kennard 1970; Friedman et al. 2010). The optimal λ value was selected through cross‐validation using the “cv.glmnet” function to obtain the best predictive performance. Coefficient profiles were generated with “plot.glmnet,” and genes retaining nonzero coefficients were considered key candidate features.

### 2.35 Protein–Protein Interaction (PPI) Network Analysis and Hub Gene Validation

A PPI network of the DEGs was generated using the STRING database (https://string‐db.org/) with a confidence score threshold of 0.7. The resulting network was imported into Cytoscape (v3.7.2) for visualization, and hub genes were screened according to node degree centrality.

### 2.36 Clustering and Heatmap Visualization

Expression data of the key genes were subjected to hierarchical clustering and heatmap visualization using the “pheatmap” package (v1.0.12) in R. Euclidean distance was used as the clustering method, and data were standardized using the *z*‐score method.

### 2.37 Immune Infiltration Analysis

Immune infiltration patterns across 22 immune cell populations were inferred from gene expression profiles using the CIBERSORT algorithm in R (v4.2.1), together with the “e1071” and “preprocessCore” packages. The results were visualized using ggplot2.

### 2.38 Cell Grouping and FOXM1 Gene Knockdown

Mouse primary aortic ECs (MAECs; MIC‐iCell‐c003) were obtained from iCell Bioscience Inc. and cultured in DMEM containing 10% FBS (Gibco, USA) and 1% penicillin–streptomycin solution (Invitrogen, USA) at 37°C in a humidified incubator with 5% CO_2_. Cells were seeded in 6‐well plates at 1 × 10^5^ cells/well and assigned to the following groups:

G0. control group: cells maintained under normal culture conditions and treated with PBS as the baseline control;

G1. oxidized low‐density lipoprotein (ox‐LDL) group: cells exposed to 200 μM ox‐LDL (H1009, Sigma‐Aldrich, USA) for 4 h, followed by an additional 24 h incubation;

G2. ox‐LDL + RSV NPs group: treated with 200 μM ox‐LDL for 4 h, followed by 50 μg/mL RSV NPs for 24 h;

G3. ox‐LDL + PM@RSV NPs group: treated with 200 μM ox‐LDL for 4 h, followed by 50 μg/mL PM@RSV NPs for 24 h;

G4. ox‐LDL + PM@RSV NPs + sh‐NC group: cells transfected with sh‐NC prior to exposure to 200 μM ox‐LDL for 4 h and subsequent treatment with 50 μg/mL PM@RSV NPs for 24 h;

G5. ox‐LDL + PM@RSV NPs + sh‐FOXM1 group: transfected with sh‐FOXM1‐2, then treated with 200 μM ox‐LDL for 4 h, followed by 50 μg/mL PM@RSV NPs for 24 h;

FOXM1 gene knockdown was achieved via shRNA transfection. The sequences of sh‐FOXM1‐1 and sh‐FOXM1‐2 were designed and synthesized by GenePharma (Shanghai, China) as follows: sh‐FOXM1‐1: GCTCCATAGAAATGTGACCAT; sh‐FOXM1‐2: CGCTACTTGACATTGGACCAA; Negative control shRNA (sh‐NC): TTCTCCGAACGTGTCACGT. Transfection was carried out using Lipofectamine RNAiMAX reagent (13,778,030, Invitrogen, USA). After 24 h, FOXM1 knockdown efficiency was confirmed by RT‐qPCR and WB analysis.

### 2.39 JC‐1 Staining for Mitochondrial Membrane Potential (MMP)

Cells were plated into 6‐well plates at 1 × 10^5^ cells/well and processed according to the experimental protocol. MMP was evaluated using a JC‐1 staining kit (C2006, Beyotime, China). After incubation with JC‐1 working solution at 37°C for 20 min, cells were rinsed twice with pre‐warmed PBS and observed under a Zeiss Axio Observer 7 microscope (Germany). The red/green fluorescence intensity ratio (aggregates/monomers) was quantified using ImageJ (v1.53) to assess MMP changes.

### 2.40 TEM for Mitochondrial Ultrastructure Observation

MAECs or CD31^+^ cells isolated by flow cytometric sorting were fixed overnight at 4°C with 2.5% glutaraldehyde (340,855, Sigma‐Aldrich). Post‐fixation was performed using 1% osmium tetroxide for 10 min. The samples were dehydrated using a graded ethanol series from 30% to 100%, embedded in low‐viscosity epoxy resin, and polymerized at 60°C for 24 h. Ultrathin sections of 70 nm were cut using a Leica UC7 ultramicrotome (Leica Microsystems, Germany), followed by double staining with 2% uranyl acetate for 10 min and 0.5% lead citrate (Ted Pella, USA) for 5 min. Mitochondrial ultrastructure was then observed and imaged using a TEM (JEM‐1400, JEOL, Japan).

### 2.41 Senescence‐Associated β‐Galactosidase (SA‐β‐Gal) Staining for Cellular Senescence

Cells were stained using a SA‐β‐Gal staining kit (C0602, Beyotime, China) at 37°C for 12 h in a CO_2_‐free environment. Positive cells (blue) were imaged using an inverted microscope, and the percentage was quantified using ImageJ. For tissue staining, mouse aortas were fixed in 4% PFA for 4 h, dehydrated in 30% sucrose (S0389, Sigma‐Aldrich, USA) for 24 h, embedded in OCT, and sectioned at 6 μm. Sections were stained identically, imaged with a Leica DM4000B microscope (Germany), and positive areas were quantified using ImageJ.

### 2.42 EdU Staining for Cell Proliferation

Cell proliferation was assessed using an EdU Cell Proliferation Kit (C0081S, Beyotime, China). Cells were incubated with EdU (10 μM) for 2 h, washed with PBS, and processed with Click‐iT reaction solution. Cell nuclei were stained with DAPI, and fluorescence images were captured for analysis. The percentage of EdU‐positive cells was subsequently determined using ImageJ software.

### 2.43 Wound Healing Assay for Repair Assessment

Confluent cell monolayers were scratched using a sterile pipette tip and rinsed with pre‐warmed PBS to remove detached cells. Cells were subsequently cultured in medium containing 0.5% FBS to minimize proliferation interference. Images of wound closure were obtained at 0 and 24 h using a Zeiss Axio Observer 7 microscope (Germany). Migration ability was evaluated by calculating wound closure rates with ImageJ software (v1.53). Each experiment was independently repeated three times, with at least five random fields analyzed per replicate.

### 2.44 Tube Formation Assay to Evaluate Angiogenic Capacity

Matrigel (356,231, Corning, USA) was coated onto 96‐well plates on ice and solidified at 37°C for 30 min. Cell suspensions were seeded at 1 × 10^4^ cells/well and incubated at 37°C with 5% CO_2_ for 6 h. Capillary‐like tubular structures were photographed using a Zeiss Axio Observer 7 microscope (Germany), and the number and total length of tubes were quantified using ImageJ.

### 2.45 Flow Cytometry for Cell Cycle Analysis

Cells were dissociated with 0.25% trypsin (Invitrogen, USA) at 37°C for approximately 5 min, washed thoroughly with pre‐warmed PBS, and resuspended in PBS. Cells were then fixed in pre‐cooled 70% ethanol overnight at 4°C. Following ethanol removal, samples were stained with PI staining solution (P4170, Sigma‐Aldrich, USA) for 30 min at ambient temperature in the dark. PI fluorescence signals were measured using a BD FACSCanto II flow cytometer (BD Biosciences, USA), and cell cycle distribution was analyzed quantitatively with ModFit LT software (v5.0).

### 2.46 Comet Assay

DNA strand damage was evaluated using a single‐cell gel electrophoresis assay with a commercial Comet Assay Kit (4250–050‐K, R&D Systems, USA). Briefly, cells were mixed with low‐melting‐point agarose (A4718, Sigma‐Aldrich, USA) and spread onto pre‐coated microscope slides. Following lysis at 4°C for 2 h, samples were subjected to alkaline electrophoresis conditions (pH > 13, 300 mA, 25 V for 20 min). After neutralization for 10 min, slides were stained with ethidium bromide (5 μg/mL; E7637, Sigma‐Aldrich, USA). DNA comet tails were visualized under a fluorescence microscope, and tail intensity was quantified using CASP 1.2.3b software (CaspLab, Poland).

### 2.47 Systemic Toxicity Evaluation

Eight‐week‐old male C57BL/6J mice were acclimatized for 1 week and randomly assigned to control, RSV NPs, and PM@RSV NPs groups (*n* = 6/group). Treatment groups received weekly tail vein injections of RSV NPs or PM@RSV NPs (10 mg/kg, 100 μL PBS) for 6 weeks; controls received PBS (100 μL). After treatment, mice were anesthetized with sodium pentobarbital (100 mg/kg, i.p.), blood was collected by cardiac puncture, and major organs (heart, liver, spleen, lung, kidney) were harvested for pathological examination.

### 2.48 Blood Biochemical Analysis

Liver and kidney functions were assessed using a biochemical analyzer (CobasBio, Roche, Switzerland). Whole blood samples from mice were centrifuged at 3000 r/min for 10 min at 4°C to isolate serum. The levels of alanine aminotransferase (ALT), aspartate aminotransferase (AST), blood urea nitrogen (BUN), and creatinine (Cr) were measured to evaluate the potential hepatic and renal toxicity of PM@RSV NPs.

### 2.49 In Vivo Biodistribution and Targeting of PM@RSV NPs

ApoE^−^/^−^ mice were acclimatized for 1 week and randomly assigned to control, RSV NPs, and PM@RSV NPs groups (*n* = 6/group). Mice were fed an HFD (21% fat, 0.15% cholesterol; D12079B, Research Diets, USA) for 2 weeks before receiving tail vein injection of DiR‐labeled RSV NPs or PM@RSV NPs (10 mg/kg, 100 μL PBS); controls received PBS. Whole‐body fluorescence was monitored at 12 h post‐injection using an IVIS Spectrum system (PerkinElmer, USA). At 24 h, mice were sacrificed, and the aortic arch, liver, and other major organs were harvested for fluorescence imaging to assess biodistribution and plaque accumulation.

### 2.50 Statistical Software and Data Analysis Methods

Statistical analyses were performed using R language (v4.2.1) within the integrated development environment RStudio. All data were processed and visualized using GraphPad Prism. Quantitative results are presented as mean ± standard deviation (Mean ± SD). Two‐group comparisons were conducted using unpaired *t*‐tests, and multiple‐group comparisons by one‐way ANOVA. Variance homogeneity was assessed by Levene's test; post hoc pairwise comparisons were performed using LSD‐t (equal variances) or Dunnett's T3 test (unequal variances). *p* < 0.05 was considered statistically significant.

## 3 Results

### 3.1 AS Accelerates EC Senescence and Induces Mitochondrial Dysfunction, Impairing Vascular Regeneration

To explore the contribution of EC senescence to AS progression, an AS model was established by feeding ApoE^−/−^ mice an HFD, followed by systematic analysis using aortic tissues and CD31^+^ ECs isolated by flow cytometry (Figure S1A).

H&E staining revealed significant intimal thickening and well‐defined plaque formation in the aortic sinus of the AS model group, indicating vascular wall remodeling accompanied by typical pathological changes. Oil Red O staining further demonstrated substantial neutral lipid deposition in the aortic wall of the AS group, confirming successful establishment of the AS model. In addition, Masson's trichrome staining demonstrated collagen fiber accumulation in the intimal region, suggesting the initiation of fibrotic remodeling within the atherosclerotic plaques (Figure S1B,C).

Compared with controls, CD31^+^ EC protein lysates from the AS model group showed markedly elevated p21 and p16 expression (Figure S1D), suggesting endothelial senescence. Consistently, dual immunofluorescence staining detected numerous CD31^+^p16^+^ double‐positive cells in the AS group, while such cells were rarely observed in controls, further confirming EC senescence (Figure S1E). To further explore the potential mechanisms underlying EC senescence, we evaluated the role of mitochondrial functional alterations during the senescence process. WB analysis of CD31^+^ ECs revealed that the mitochondrial fusion proteins OPA1 and MFN2 were significantly downregulated in the AS model group, whereas the fission‐related proteins DRP1 and FIS1 were markedly upregulated (Figure S1F), indicating a disruption of mitochondrial dynamics and a shift toward mitochondrial fission. Mitochondrial fragmentation is associated with cellular senescence, apoptosis, and metabolic dysfunction. MitoSOX staining of CD31^+^ ECs was performed to detect mitochondrial ROS levels. Flow cytometric analysis of MitoSOX‐stained CD31^+^ ECs revealed significantly elevated mitochondrial ROS levels in the AS model group (Figure S1G,H). Excessive ROS accumulation damages mitochondrial DNA (mtDNA), triggers apoptosis, and activates senescence‐related signaling pathways, further accelerating the progression of AS. Moreover, ATP production in CD31^+^ ECs was significantly reduced in the AS model group (Figure S1I), indicating impaired mitochondrial function and diminished energy metabolism, which may further compromise the ability of ECs to maintain vascular integrity and repair injury. These findings indicate that disrupted mitochondrial dynamics, excessive oxidative stress, and defective energy metabolism contribute importantly to EC senescence and may further promote AS progression.

To assess vascular repair and regeneration capacity changes, we examined the expression of angiogenesis‐related markers CD31 and VEGF in aortic tissues. The results showed significant downregulation of CD31 and VEGF expression in aortic tissues from the model group (Figure S1J), indicating impaired vascular regeneration. The decline in EC proliferation and disruption of angiogenic signaling hindered effective vascular repair, potentially accelerating plaque formation and further deteriorating the ischemic microenvironment within the lesion area.

Moreover, enhanced oxidative stress and inflammatory responses further exacerbated vascular dysfunction. In the AS model group, ECs exhibited significantly reduced levels of the antioxidant enzymes SOD and CAT, accompanied by a marked increase in the lipid peroxidation product MDA (Figure S1K), indicating impaired antioxidant capacity and increased oxidative stress in ECs. Concurrently, pro‐inflammatory cytokines TNF‐α and IL‐6 were significantly upregulated in ECs (Figure S1L), suggesting that activation of inflammatory signaling during AS lesion development may have further aggravated EC injury. Oxidative stress and inflammation likely formed a vicious cycle, deteriorating the vascular microenvironment and accelerating lesion progression.

During AS, EC senescence, mitochondrial dysfunction, and impaired vascular repair formed a self‐reinforcing pathological loop. This vicious cycle not only accelerated vascular lesion development but also severely limited the intrinsic repair capacity of the vasculature, ultimately contributing to plaque instability and the continued progression of AS. Current therapeutic strategies remain insufficient in suppressing endothelial senescence and enhancing vascular regeneration. Therefore, innovative therapeutic approaches targeting mitochondrial dysfunction, reducing oxidative stress, and inhibiting senescence‐associated molecular pathways may offer new opportunities for treating AS.

### 3.2 Successful Preparation and Characterization of PM@RSV NPs

During the progression of AS, EC senescence and mitochondrial dysfunction lead to impaired vascular regeneration, thereby exacerbating lesion development. RSV was selected as a candidate therapeutic agent due to its antioxidant, anti‐inflammatory, and pro‐angiogenic properties. However, its low bioavailability limits its therapeutic efficacy. To address this, PM@RSV NPs were developed. By leveraging platelet‐membrane adhesion proteins and immune‐evasive properties, this system aimed to enhance RSV stability, improve preferential delivery to injured ECs and AS lesion‐associated vascular endothelium, and thereby improve the pathological features of AS.

**FIGURE 1:**
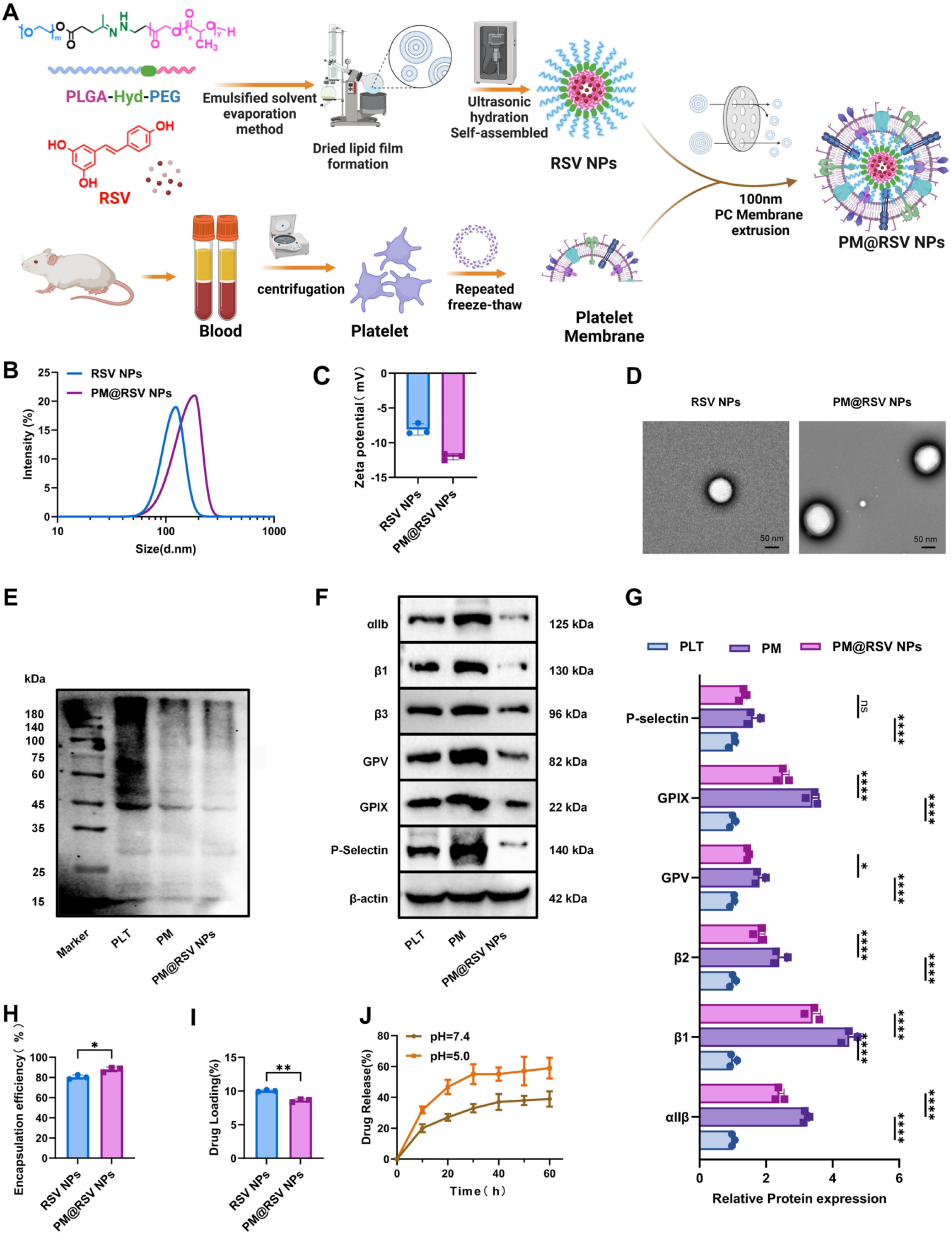
Preparation and characterization of the PM@RSV NPs. (A) Schematic diagram of PM@RSV NPs preparation; (B) Particle size distribution detected by DLS; (C) Zeta potential measured by DLS; (D) TEM image of nanoparticle morphology, bar = 50 nm; (E) SDS‐PAGE analysis of protein composition in PLT, PM, and PM@RSV NPs; (F, G) Western blot detection of PM‐specific adhesion proteins αIIb, β1, β3, GPV, GPIX, and P‐selectin in PM and PM@RSV NPs; (H, I) HPLC analysis of drug encapsulation efficiency and drug loading capacity of PM@RSV NPs; (J) Drug release profiles of PM@RSV NPs under different pH conditions. Experiments were repeated three times. ns indicates no significant difference between groups; *p < 0.05, **p < 0.01, ****p < 0.0001.

PM@RSV NPs were successfully fabricated using a solvent evaporation method followed by a membrane‐coating technique (Figure 1A). DLS analysis revealed that PM@RSV NPs had an average particle size of 172.2 nm and a surface zeta potential of −19.3 mV, indicating good dispersibility and surface stability (Figure 1B,C). TEM revealed that the nanoparticles were uniformly spherical and were entirely coated with an intact PM (Figure 1D). These results confirmed the successful construction of structurally well‐defined PM@RSV NPs.

To confirm successful PM coating, WB analysis was conduct to examine PM‐specific adhesion proteins. Most proteins from the original PLT membrane were retained on the PM surface, and these proteins were also preserved in the PM@RSV NPs after coating, without significant loss (Figure 1E). Quantitative analysis showed that membrane receptors and transmembrane proteins such as GPV, GPIX, and P‐selectin were highly expressed in both the PM and PM@RSV NPs (Figure 1F,G). These results further confirmed the successful surface modification of the nanoparticles with the PM.

HPLC analysis revealed that PM@RSV NPs exhibited an RSV encapsulation efficiency of 89.6% and a drug loading capacity of 8.31% (Figure 1H,I). Drug release studies performed under different pH environments showed that PM@RSV NPs exhibited a markedly faster RSV release profile under acidic conditions (pH 5.0) compared with physiological conditions (Figure 1J). These findings indicate that PM@RSV NPs possess favorable pH‐responsive drug release properties, facilitating targeted drug delivery to diseased sites.

The stability of nanomaterials is critical for their application. To assess both short‐term and long‐term storage stability, DLS was used to monitor changes in particle size and zeta potential (Figure S2A). DLS results showed that after 24 h of storage at 37°C, the average particle size and zeta potential of PM@RSV NPs remained stable without significant variation (Figure S2B,C), indicating good short‐term physical stability. Moreover, after 14 days of storage at 4°C, the nanoparticles maintained consistent particle size and zeta potential under both conditions (Figure S2D,E). These data further confirmed that PM@RSV NPs exhibited excellent physical stability during both short‐ and long‐term storage, supporting their potential for further application.

To evaluate the biocompatibility of PM@RSV NPs in ECs, MAECs were subjected to dose‐dependent toxicity assays (Figure S3A). Cell viability was evaluated by the CCK‐8 assay. No significant differences were observed between the untreated group and the low‐ (10 μg/mL) or medium‐dose (25 μg/mL) PM@RSV NPs groups, whereas a modest reduction in viability was detected at the high dose (50 μg/mL), although overall viability remained relatively high (Figure S3B). Consistently, Calcein‐AM/PI staining showed preserved cell viability in the low‐ and medium‐dose groups, while the high‐dose group exhibited a slight increase in PI‐positive cells (Figure S3C,D). Flow cytometric analysis of apoptosis indicated no significant change in apoptotic rates at low and medium doses and a slight but statistically insignificant increase at the high dose (Figure S3E,F). These findings suggest that PM@RSV NPs exhibited no apparent cytotoxicity within physiologically relevant dose ranges, indicating favorable cellular biocompatibility based on viability and apoptosis readouts and providing a foundation for in vivo studies.

The above results confirmed that PM@RSV NPs were successfully prepared and exhibited good biocompatibility within the tested dosage range, with no significant cytotoxicity toward ECs.

### 3.3 PM@RSV NPs Target Damaged ECs

To investigate the effect of PM modification on the cellular uptake of PM@RSV NPs in ECs, confocal microscopy and flow cytometry were employed to systematically analyze nanoparticle internalization (Figure S4A). Flow cytometry revealed that the cellular uptake of PM@RSV NPs was significantly higher than that of uncoated RSV NPs at 1 and 4 h (Figure S4B), indicating that PM coating effectively enhanced nanoparticle internalization. Confocal fluorescence imaging further demonstrated that Cy5‐labeled PM@RSV NPs predominantly co‐localized with the lysosomal marker LAMP1 within cells (Figure S4C), suggesting that the nanoparticles entered cells primarily via the endosome‐lysosome pathway rather than transmembrane diffusion or other uptake mechanisms.

In an ox‐LDL‐induced endothelial injury model, the uptake of PM@RSV NPs was further evaluated in both normal MAECs and ox‐LDL‐induced injured MAECs. The results showed that ox‐LDL‐induced injured MAECs exhibited significantly higher uptake of PM@RSV NPs compared with normal MAECs (Figure S4D), indicating that the injured endothelial microenvironment facilitated preferential nanoparticle accumulation. This phenomenon may be associated with inflammatory endothelial adhesion pathways on injured MAECs and with platelet‐membrane adhesion proteins retained on PM@RSV NPs. Specifically, PM‐derived GPV and P‐selectin were retained on the nanoparticle surface and could strengthen nanoparticle‐cell interactions with injured ECs. To confirm whether the targeted uptake of PM@RSV NPs was mediated by key ligands derived from the PM, Cy5‐labeled PM@RSV NPs were preincubated with neutralizing antibodies against GPV and P‐selectin to block PM‐derived adhesion ligands, followed by flow cytometric quantification of nanoparticle uptake (Figure S4E). The results showed that antibody pretreatment significantly reduced PM@RSV NP internalization, demonstrating that GPV and P‐selectin played critical roles in the targeted uptake of PM@RSV NPs by ox‐LDL‐injured MAECs. These findings confirmed that PM modification endowed PM@RSV NPs with bio‐recognition capabilities, allowing them to accumulate in damaged endothelial environments preferentially.

In summary, PM modification enhanced the overall uptake efficiency of PM@RSV NPs and imparted specific recognition of ox‐LDL‐injured MAECs. This process mainly depended on platelet‐membrane adhesion proteins, particularly GPV and P‐selectin, rather than on a nonspecific increase in nanoparticle uptake. With this targeting capability, PM@RSV NPs exhibit significant potential for precision drug delivery and therapeutic intervention in cardiovascular diseases, offering new avenues for nanotechnology‐based cardiovascular treatments.

### 3.4 Transcriptomic Profiling Reveals the Key Role of FOXM1 Upregulation by PM@RSV NPs

**FIGURE 2:**
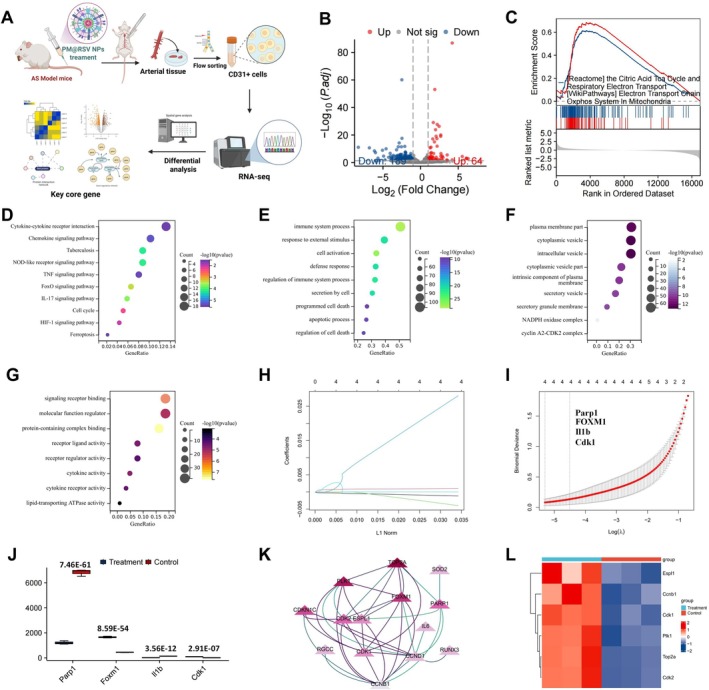
Transcriptomic analysis revealed the critical role of FOXM1 in the mechanism of PM@RSV NPs. (A) Schematic diagram of the RNA‐seq workflow (Created in BioRender); (B) Volcano plot of DEGs; (C) GSEA showing gene set enrichment; (D) KEGG pathway analysis indicating functional pathways enriched by DEGs; (E, G) GO enrichment analysis of DEGs in terms of BP (E), CC (F), and MF (G); (H) LASSO coefficient distribution of DEGs; (I) Selection of the optimal parameter (lambda) in the LASSO model; (J) Boxplots of expression levels of the four key features identified by LASSO; (K) PPI network showing interactions between FOXM1 and core genes; (L) Clustering heatmap of core gene expression across different sample groups. Control: *N* = 3; Treatment: *N* = 3.

To further investigate the molecular mechanisms of PM@RSV NPs in AS, RNA‐seq was performed on CD31^+^ ECs sorted by flow cytometry from the arterial tissues of PM@RSV NPs‐treated ApoE^−/−^ mice and AS model control ApoE^−/−^ mice, generating a comprehensive gene expression profile (Figure 2A). A total of 253 DEGs were detected, including 64 upregulated and 189 downregulated genes (Figure 2B), indicating that PM@RSV NPs induced widespread transcriptional regulation at the gene level.

GSEA showed that these DEGs were mainly enriched in mitochondrial energy metabolism‐related pathways, including the TCA cycle, respiratory electron transport, and oxidative phosphorylation (Figure 2C), suggesting that PM@RSV NPs may enhance EC vitality by improving mitochondrial energy metabolism. Consistently, KEGG pathway analysis revealed enrichment in several key signaling pathways, including the FoxO and IL‐17 signaling pathways, cell cycle regulation, and ferroptosis (Figure 2D), indicating the potential involvement of PM@RSV NPs in inflammation modulation, cell proliferation, and metabolic homeostasis. GO analysis further supported these findings. In the BP category, DEGs were enriched in immune system processes, cell activation, and regulation of cell death. In the CC category, they were enriched in plasma membrane components, NADPH oxidase complexes, and cyclin A2‐CDK2 complexes. In the MF category, significant enrichment was observed in signaling receptor binding, receptor‐ligand activity, and lipid‐transporting ATPase activity (Figure 2E–G). These results suggest that PM@RSV NPs may improve arterial function by modulating metabolic pathways, immune responses, and cell cycle‐related signaling networks.

To screen potential core targets, LASSO regression was applied to the 253 DEGs, and the optimal λ value was selected by 10‐fold cross‐validation (Figure 2H). Four feature genes were identified: PARP1, IL1B, FOXM1, and CDK1 (Figure 2I). Among these, PARP1 and IL1B were significantly downregulated in the PM@RSV NPs treatment group, whereas FOXM1 and CDK1 were significantly upregulated (Figure 2J). Notably, FOXM1 plays a pivotal role in cell proliferation, G2/M phase transition, and DNA damage repair (Khan et al. 2023), and is closely linked to the FoxO pathway (Zhao and Lam 2012). Furthermore, previous studies have shown that RSV can upregulate FOXM1 expression (Poudel et al. 2022), consistent with our findings. Therefore, FOXM1 may serve as a central mediator of the protective effects of PM@RSV NPs against AS.

A PPI network was constructed to elucidate the regulatory network of FOXM1 further. The results revealed that FOXM1 functioned as a central hub, forming tight interactions with key genes such as PLK1, TOP2A, ESPL1, CDK2, CCNB1, and CDK1 (Figure 2K). These genes are strongly associated with cell cycle regulation and cell proliferation (Radulovic et al. 2010; Wang et al. 2025), suggesting that PM@RSV NPs may enhance endothelial regeneration by modulating FOXM1, thereby improving arterial repair. Cluster heatmap analysis further showed that the expression levels of these key genes were markedly upregulated in the PM@RSV NPs treatment group (Figure 2L), consistent with phenotypic improvements in endothelial function, thus reinforcing the central regulatory role of FOXM1.

Considering the close association between DEGs and inflammatory regulation, CIBERSORT was used to evaluate the infiltration patterns of 22 immune cell types between groups. Naïve B cells were significantly decreased after PM@RSV NP treatment (Figure S5A,B). Moreover, FOXM1 expression was negatively correlated with naïve B cells, resting dendritic cells, and resting NK cells (Figure S5C). These findings suggest that PM@RSV NPs may promote EC proliferation and repair by regulating FOXM1 and modulating the immune microenvironment. Specifically, the treatment appears to suppress the aberrant activation of B cells and dendritic cells, thereby contributing to the amelioration of the pathological progression of AS.

Overall, RNA‐seq analysis revealed the gene regulatory landscape induced by PM@RSV NPs in arterial ECs and identified FOXM1 as a key regulatory factor. FOXM1 may contribute to endothelial proliferation, cell cycle progression, and immune microenvironment optimization. These findings provide mechanistic evidence for the therapeutic role of PM@RSV NPs in AS and support FOXM1 as a potential target for future intervention strategies.

### 3.5 PM@RSV NPs Activate FOXM1 to Improve Mitochondrial Dysfunction

**FIGURE 3:**
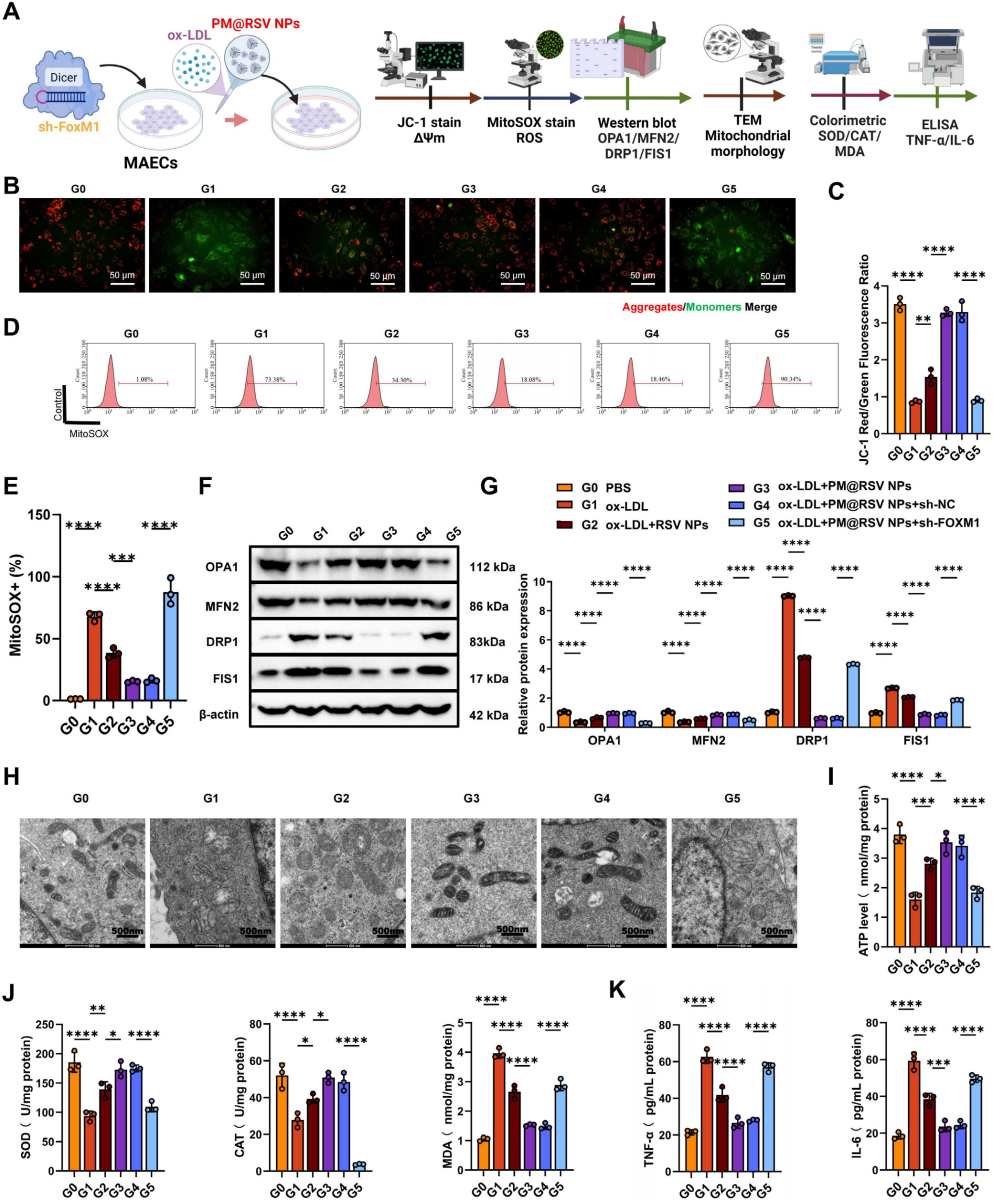
PM@RSV NPs improved mitochondrial dysfunction by activating FOXM1. (A) Schematic diagram of the experimental procedure verifying the mechanism by which PM@RSV NPs activate FOXM1 to improve mitochondrial function (Created in BioRender); (B, C) JC‐1 staining to detect changes in MMP (red/green fluorescence ratio) in MAECs, bar = 50 μm; (D, E) Flow cytometric analysis of mitochondrial ROS levels in MAECs using the MitoSOX fluorescent probe staining; (F, G) Western blot analysis of mitochondrial fusion proteins OPA1 and MFN2 and fission proteins DRP1 and FIS1 in MAECs; (H) TEM observation of mitochondrial ultrastructure, bar = 500 nm; (I) ATP assay to detect cellular ATP production; (J) Levels of SOD, CAT, and MDA in ECs; (K) ELISA detection of pro‐inflammatory cytokines TNF‐α and IL‐6 in ECs. All experiments were repeated three times. *p < 0.05, **p < 0.01, ***p < 0.001, ****p < 0.0001 between groups.

To further elucidate the mechanism by which PM@RSV NPs improve mitochondrial function via FOXM1 activation, an ox‐LDL‐induced EC injury model was established and treated with PM@RSV NPs and FOXM1 gene interference, respectively (Figure 3A).

To verify the role of FOXM1 in PM@RSV NPs‐mediated mitochondrial function restoration, two FOXM1‐specific shRNAs (sh‐FOXM1‐1 and sh‐FOXM1‐2) were designed and screened. RT‐qPCR and WB analyses showed that sh‐FOXM1‐2 exhibited higher knockdown efficiency at both mRNA and protein levels and was therefore selected for subsequent experiments (Figure S6A–C). In an ox‐LDL‐induced EC injury model, PM@RSV NPs significantly upregulated FOXM1 mRNA and protein expression, while FOXM1 knockdown completely reversed this upregulation (Figure S6D–F), suggesting that the effects of PM@RSV NPs are dependent on FOXM1 activation.

JC‐1 staining results revealed that ox‐LDL treatment significantly reduced MMP, whereas both RSV NPs and PM@RSV NPs partially restored MMP. PM@RSV NPs exhibited a more pronounced recovery effect, which was significantly weakened upon FOXM1 knockdown, indicating that PM@RSV NPs maintained mitochondrial homeostasis through FOXM1 (Figure 3B,C). Further flow cytometric analysis using the MitoSOX staining demonstrated that PM@RSV NPs significantly reduced mitochondrial ROS levels, while ROS levels were markedly increased following FOXM1 knockdown (Figure 3D,E), suggesting that FOXM1 may suppress oxidative stress damage by regulating antioxidant mechanisms.

WB analysis was further conducted to evaluate proteins associated with mitochondrial dynamics. Compared with the ox‐LDL group, RSV NPs upregulated the mitochondrial fusion proteins OPA1 and MFN2, while downregulating the fission proteins DRP1 and FIS1 (Figure 3F,G). PM@RSV NPs exhibited a stronger effect in modulating the fusion/fission balance. These regulatory effects on protein expression were completely reversed following FOXM1 knockdown, indicating that PM@RSV NPs promote mitochondrial fusion and inhibit excessive fission via FOXM1, thereby maintaining mitochondrial homeostasis. TEM further confirmed these findings; PM@RSV NPs treatment significantly improved mitochondrial ultrastructure, restoring intact cristae, whereas FOXM1 knockdown aggravated mitochondrial damage and structural disruption (Figure 3H).

To determine whether PM@RSV NPs improve mitochondrial metabolic function, intracellular ATP production levels were measured. Quantitative analysis showed that ATP levels increased significantly after RSV NP treatment compared with the ox‐LDL group and were further elevated in the PM@RSV NP group. However, this effect was markedly weakened following FOXM1 knockdown (Figure 3I), indicating that FOXM1 plays a key regulatory role in PM@RSV NPs‐mediated energy metabolism restoration.

Additionally, to evaluate the antioxidant defense effects of PM@RSV NPs, the activities of antioxidant enzymes SOD and CAT, along with the level of the lipid peroxidation marker MDA, were measured. Ox‐LDL treatment significantly reduced SOD and CAT activities while increasing MDA levels. Both RSV NPs and PM@RSV NPs effectively alleviated this oxidative stress, with PM@RSV NPs showing a more pronounced effect (Figure 3J). The antioxidant effects were significantly weakened upon FOXM1 knockdown, indicating that FOXM1 plays a central role in regulating oxidative stress balance.

Finally, ELISA was used to determine TNF‐α and IL‐6 levels and evaluate the inflammatory regulatory effect of PM@RSV NPs. Ox‐LDL stimulation markedly increased TNF‐α and IL‐6 expression, whereas RSV NPs and PM@RSV NPs significantly attenuated this response, with PM@RSV NPs exerting the strongest inhibitory effect (Figure 3K). After FOXM1 knockdown, both cytokines were increased again, further indicating that PM@RSV NPs suppress ox‐LDL‐induced endothelial inflammation in a FOXM1‐dependent manner.

In summary, PM@RSV NPs significantly ameliorated mitochondrial dysfunction by upregulating FOXM1, as evidenced by restored MMP, reduced ROS levels, modulation of mitochondrial dynamics, enhanced ATP production, increased antioxidant enzyme activity, and suppression of inflammatory responses. These protective effects were completely abolished by FOXM1 knockdown, indicating that FOXM1 is a key regulatory factor mediating the mitochondrial protective effects of PM@RSV NPs.

### 3.6 PM@RSV NPs Inhibit EC Senescence and Promote Regeneration via FOXM1

**FIGURE 4:**
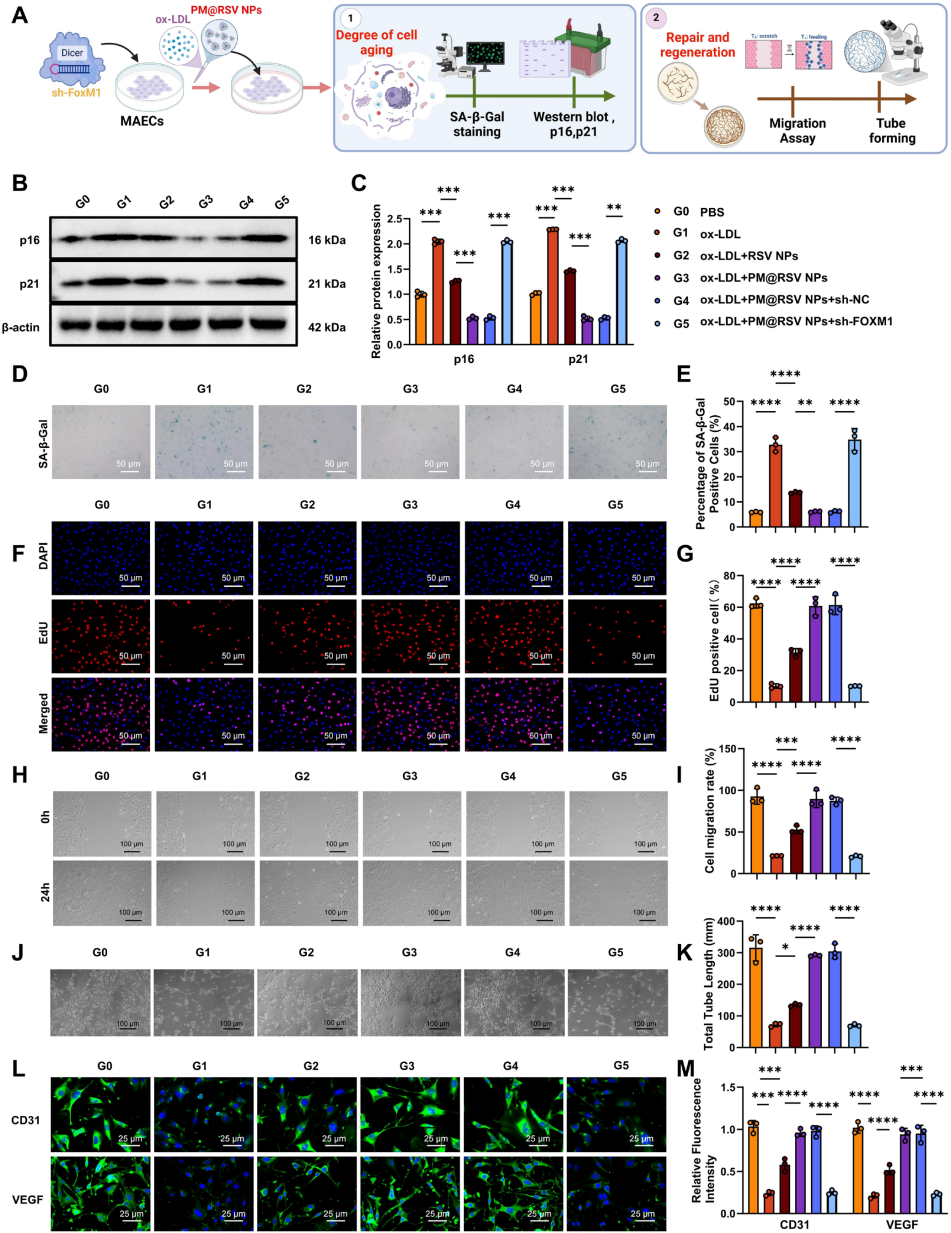
PM@RSV NPs inhibited EC senescence and promoted regeneration via FOXM1. (A) Schematic diagram of the experimental workflow illustrating the anti‐senescence and regenerative mechanisms of PM@RSV NPs in ECs (Created in BioRender); (B, C) Western blot analysis of p16 and p21 protein expression levels; (D, E) SA‐β‐Gal staining to evaluate cellular senescence, bar = 50 μm; (F, G) EdU staining to assess cell proliferation, bar = 50 μm; (H, I) Wound healing assay to analyze the effect of PM@RSV NPs on cell migration and repair capacity, bar = 100 μm; (J, K) Tube formation assay to evaluate the regulatory effect of PM@RSV NPs on angiogenesis, bar = 100 μm; (L, M) Immunofluorescence detection of angiogenesis‐related markers CD31 and VEGF, bar = 25 μm. Cell experiments were performed in triplicate. *p < 0.05, **p < 0.01, ***p < 0.001, ****p < 0.0001.

The role of PM@RSV NPs in suppressing EC senescence and promoting regeneration through FOXM1 was systematically evaluated. During senescence, ECs exhibit growth arrest, upregulation of senescence‐associated genes, and diminished regenerative capacity—changes that directly impair vascular function and accelerate the onset and progression of cardiovascular diseases (You et al. 2023). To investigate the anti‐senescence and regenerative mechanisms mediated by PM@RSV NPs, markers of cellular senescence, EC proliferation, and vascular regeneration were assessed (Figure 4A).

WB analysis showed that PM@RSV NPs significantly reduced senescence‐associated proteins p16 and p21 expression levels. This inhibitory effect was markedly reversed upon FOXM1 knockdown (Figure 4B,C), suggesting that PM@RSV NPs suppress EC senescence by downregulating p16 and p21 via FOXM1. Further SA‐β‐Gal staining revealed that, compared with the ox‐LDL group, the proportion of SA‐β‐Gal‐positive cells was significantly reduced in the RSV NPs group and further decreased in the PM@RSV NPs group, indicating effective inhibition of senescent cell accumulation. In contrast, FOXM1 knockdown markedly restored SA‐β‐Gal positivity, confirming that FOXM1 contributes to the anti‐senescence effect of PM@RSV NPs (Figure 4D,E). EdU staining showed that PM@RSV NP treatment significantly increased the proportion of EdU‐positive cells, indicating enhanced DNA synthesis and cell proliferation. However, this pro‐proliferative effect was notably weakened after FOXM1 silencing, further supporting the critical involvement of FOXM1 in PM@RSV NP‐induced endothelial proliferation (Figure 4F,G). Additionally, wound healing assays demonstrated that, compared with the ox‐LDL group, cells treated with PM@RSV NPs displayed significantly accelerated scratch closure, suggesting enhanced EC migration and injury repair. This effect was notably diminished upon FOXM1 knockdown (Figure 4H,I).

To further validate the effect of PM@RSV NPs on vascular regeneration, a tube formation assay was conducted to evaluate their role in neovascularization. The results demonstrated that PM@RSV NPs significantly promoted the formation of capillary‐like structures, whereas this effect was markedly attenuated following FOXM1 knockdown (Figure 4J,K), suggesting that PM@RSV NPs may facilitate angiogenesis through FOXM1. Immunofluorescence analysis of CD31 and VEGF further confirmed this finding; expression levels of both CD31 and VEGF were significantly upregulated following PM@RSV NPs treatment, whereas FOXM1 knockdown substantially diminished this effect (Figure 4L,M), indicating that PM@RSV NPs may enhance angiogenic signaling pathways and EC angiogenesis via FOXM1.

**FIGURE 5:**
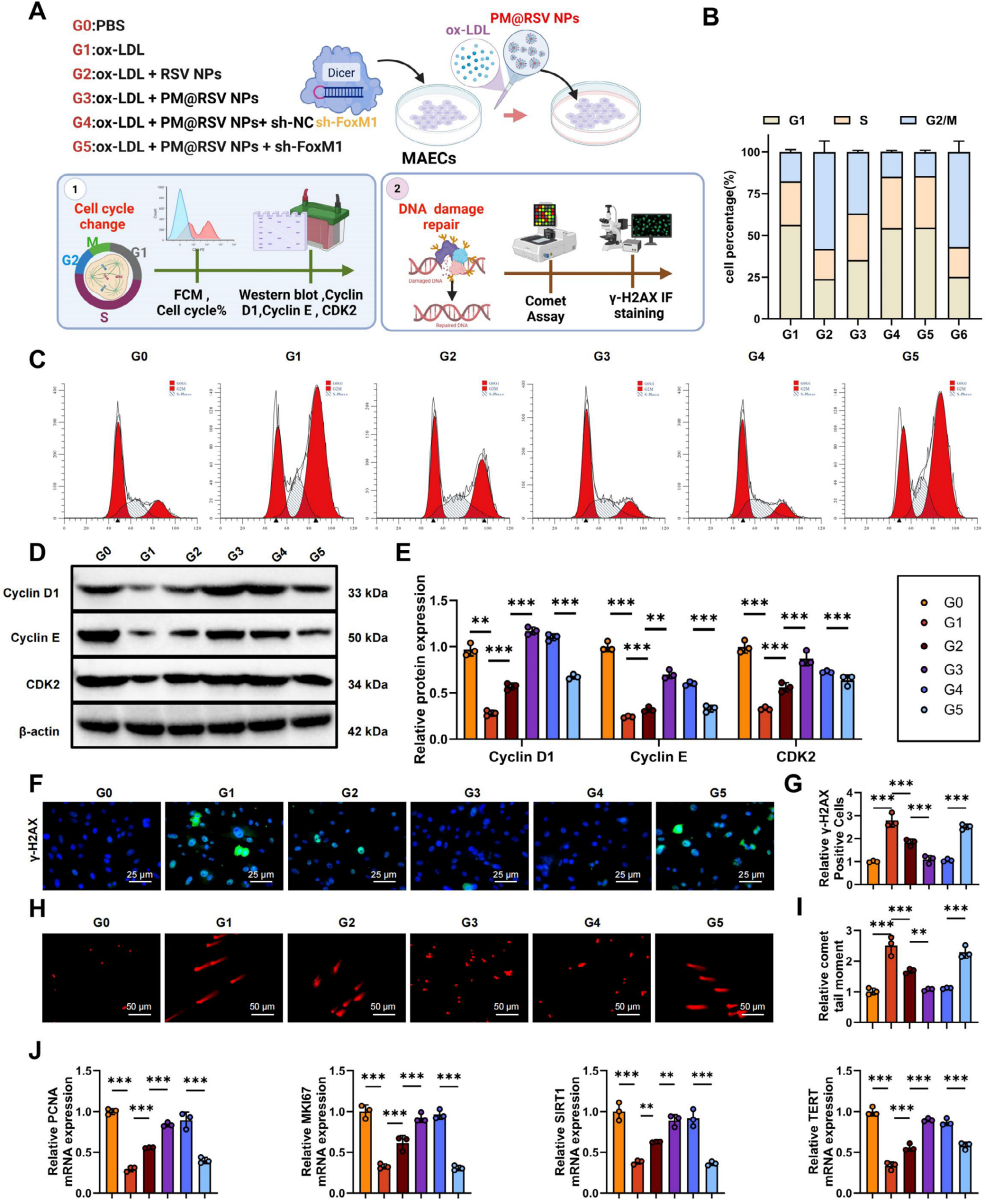
PM@RSV NPs activated cell cycle progression and DNA damage repair via FOXM1. (A) Schematic diagram of the experimental workflow illustrating the role of PM@RSV NPs in regulating the cell cycle, DNA repair, and cell regeneration (Created in BioRender); (B, C) Flow cytometry analysis of the proportion of cells in G0/G1 and S phases after PM@RSV NPs treatment; (D, E) Western blot analysis of Cyclin D1, Cyclin E, and CDK2 protein expression levels; (F, G) Immunofluorescence detection of γ‐H2AX expression to assess DNA damage, bar = 25 μm; (H, I) Comet assay analysis of DNA tail length to evaluate DNA repair capacity, bar = 50 μm; (J) RT‐qPCR analysis of mRNA expression levels of PCNA, MKI67, SIRT1, and TERT. Cell experiments were performed in triplicate. **p < 0.01, ***p < 0.001.

During cellular senescence, cell cycle arrest and accumulation of DNA damage are hallmark features, closely associated with impaired proliferative capacity and defective DNA repair mechanisms. To investigate the FOXM1‐mediated anti‐senescence mechanism of PM@RSV NPs, we evaluated cell cycle progression, DNA damage repair, and regenerative capacity (Figure 5A). Flow cytometric analysis demonstrated that PM@RSV NP treatment increased the proportion of cells transitioning from the G0/G1 phase into the S phase compared with the ox‐LDL group, suggesting enhanced cell cycle progression. In contrast, FOXM1 knockdown markedly reversed these effects and induced cell cycle arrest (Figure 5B,C). WB analysis further confirmed that PM@RSV NPs treatment markedly upregulated the expression of cell cycle regulatory proteins Cyclin D1, Cyclin E, and CDK2, whereas FOXM1 knockdown led to a substantial decrease in their expression (Figure 5D,E), indicating that PM@RSV NPs may enhance cell proliferation by promoting G1/S phase transition through FOXM1 activation.

Altered DNA repair capacity is a key feature of functional decline in senescent cells. To evaluate the effect of PM@RSV NPs on DNA damage repair, γ‐H2AX immunofluorescence staining and comet assays were performed to assess DNA damage and strand breaks. Treatment with PM@RSV NPs markedly decreased the percentage of γ‐H2AX‐positive cells, suggesting alleviation of DNA damage (Figure 5F,G). The comet assay further confirmed enhanced DNA repair, as PM@RSV NPs significantly reduced DNA tail length, suggesting reduced DNA damage (Figure 5H,I). Following FOXM1 knockdown, the proportion of γ‐H2AX‐positive cells increased, and DNA tail length significantly extended, indicating that FOXM1 may contribute to genomic stability by promoting DNA damage repair.

The decline in regenerative capacity is a central factor underlying functional deterioration during senescence. RT‐qPCR results showed that PM@RSV NPs significantly upregulated the expression of proliferation markers PCNA and MKI67, as well as DNA repair and longevity‐associated genes SIRT1 and TERT. Upon FOXM1 knockdown, the expression levels of PCNA, MKI67, SIRT1, and TERT were significantly reduced, confirming that FOXM1 mediated the PM@RSV NP‐induced regenerative and anti‐senescence effects (Figure 5J).

PM@RSV NPs may exert anti‐senescence and regenerative effects by targeting the key transcription factor FOXM1. This factor coordinately regulates cell cycle progression and DNA damage repair, thereby inhibiting EC senescence and promoting regenerative capacity.

### 3.7 In Vivo Experiments Demonstrate That PM@RSV NPs Possess Good Biosafety and Targeting Capability

To systematically evaluate the in vivo biosafety of PM@RSV NPs, healthy C57BL/6J mice were treated with PBS, RSV NPs, or PM@RSV NPs, followed by histological examination of major organs and blood biochemical analysis after 6 weeks of administration (Figure S7A). H&E staining revealed that the tissue structures of major organs, including the heart, lungs, liver, spleen, kidneys, and pancreas, remained intact in all groups, with no observable inflammation, tissue necrosis, or structural disruption (Figure S7B), indicating no significant histological differences in acute toxicity among the three groups.

Serum biochemical assays were performed to evaluate hepatic and renal function through measurements of ALT, AST, BUN, and Cr levels. Compared with the PBS control group, neither the RSV NPs group nor the PM@RSV NPs group showed significant alterations (ns), and all indicators remained within the normal physiological range (Figure S7C). These findings demonstrate that PM@RSV NPs did not induce noticeable hepatic or renal toxicity under long‐term administration, suggesting good biocompatibility and systemic safety.

To verify the targeting ability of PM@RSV NPs, an ApoE^−/−^ mouse model of AS was established. Mice were treated with DiR‐labeled PM@RSV NPs, DiR‐labeled RSV NPs, or PBS, and the biodistribution of the nanoparticles was monitored using in vivo fluorescence imaging (Figure S8A). Fluorescence imaging revealed that DiR‐labeled PM@RSV NPs predominantly accumulated in AS lesions, particularly in the heart and aortic regions, where the strongest fluorescence signals were detected. In contrast, the RSV NPs group exhibited weaker accumulation, and the PBS group showed almost no detectable signal (Figure S8B). In major reticuloendothelial system (RES)‐associated organs such as the liver, lungs, spleen, and kidneys, PM@RSV NPs exhibited relatively limited fluorescence distribution, indicating favorable targeting capability and minimal nonspecific accumulation.

Ex vivo fluorescence imaging of the entire aorta (Figure S8C) and quantitative analysis (Figure S8D) further confirmed that PM@RSV NPs produced markedly stronger aortic fluorescence signals than RSV NPs, indicating enhanced lesion‐specific accumulation. Consistently, the mean fluorescence intensity in the aorta was significantly higher in the PM@RSV NP group.

Moreover, confocal laser scanning microscopy (CLSM) was used to examine aortic root tissue sections. Results showed that in the AS plaque regions, DiR signals in the PM@RSV NPs group strongly co‐localized with the endothelial marker CD31 (Figure S8E). In contrast, co‐localization in the RSV NPs group was weaker, and the PBS group showed almost no signal, further confirming the strong ECs‐targeting ability of PM@RSV NPs. These findings support that PM@RSV NPs not only exhibit prolonged circulation in vivo but also accurately identify ECs, ensuring drug accumulation at lesion sites and reducing off‐target distribution, thereby enhancing therapeutic efficacy while minimizing systemic side effects.

Collectively, these results demonstrate that PM@RSV NPs exhibit good in vivo biosafety and low systemic toxicity, with no signs of tissue damage or liver/kidney dysfunction in major organs. Furthermore, the nanoparticles showed high accumulation in AS lesions, indicating effective endothelial targeting. The combination of in vivo imaging and immunofluorescence results suggests that platelet membrane modification enhances the lesion‐targeting capacity of the nanosystem. The favorable biodistribution profile and safety characteristics of this system provide preliminary evidence for its potential application in the treatment of vascular diseases.

### 3.8 PM@RSV NPs Delay AS Lesion Progression by Improving Mitochondrial Function Through Activation of FOXM1

**FIGURE 6:**
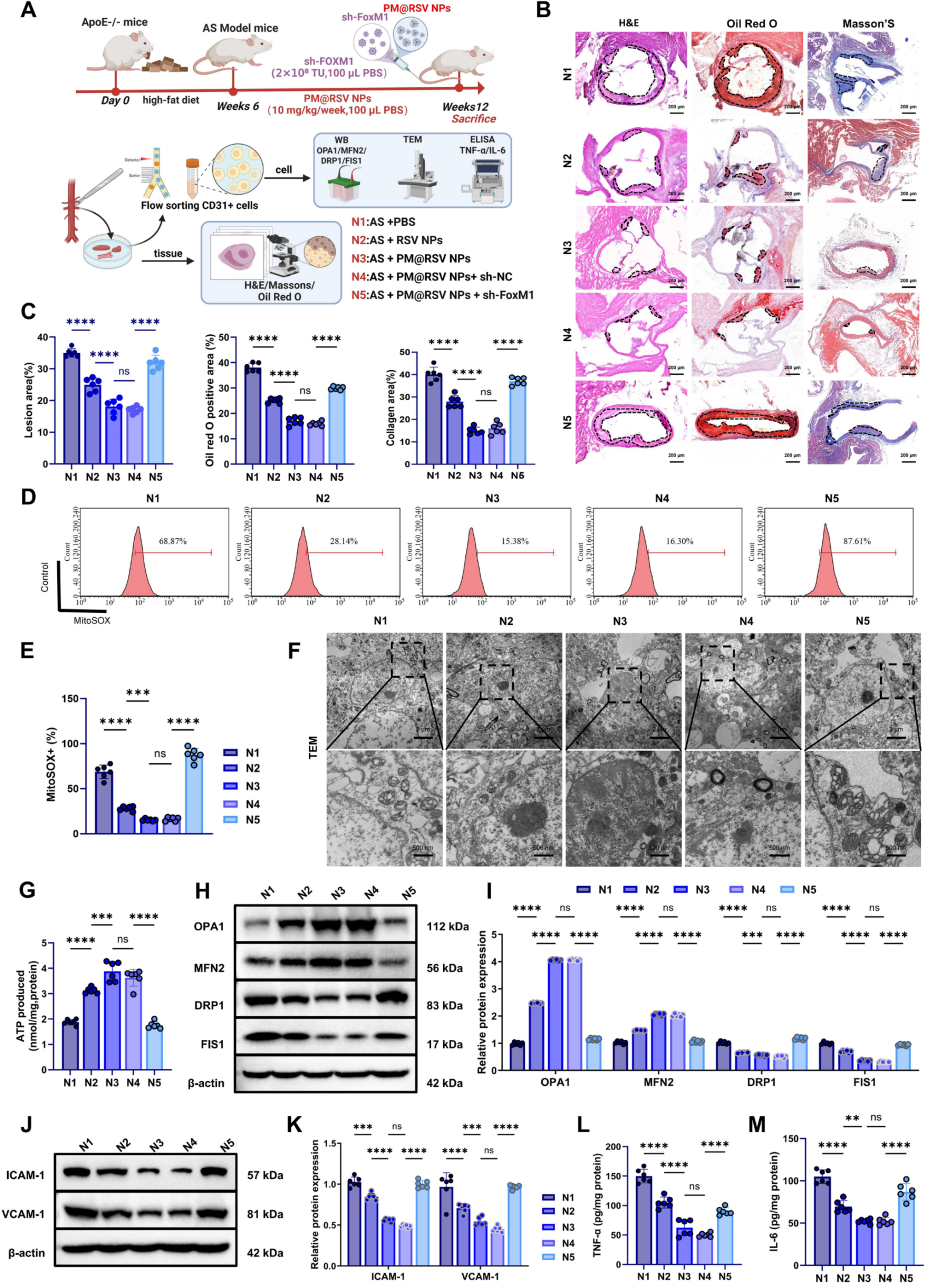
PM@RSV NPs delayed AS lesion progression by improving mitochondrial function via FOXM1 activation. (A) Schematic diagram of the experimental procedure for investigating the mechanism by which PM@RSV NPs activate FOXM1 to improve mitochondrial function and delay AS lesion progression; (B) H&E staining, Oil Red O staining, and Masson's trichrome staining of the aortic sinus sections, bar = 200 μm; (C) Quantification of plaque formation, lipid deposition, and fibrous tissue accumulation in aortic tissues; (D, E) Flow cytometric detection of mitochondrial ROS levels in aortic CD31^+^ ECs using the MitoSOX fluorescent probe staining; (F) TEM observation of mitochondrial ultrastructure in CD31^+^ aortic ECs, bar = 2 μm/500 nm; (G) ATP assay to assess ATP production in aortic CD31^+^ ECs; (H, I) Western blot analysis of the expression of OPA1, MFN2, DRP1, and FIS1 in CD31^+^ aortic ECs; (J, K) Western blot analysis of ICAM‐1 and VCAM‐1 protein expression in aortic CD31^+^ ECs; (L‐M) ELISA detection of TNF‐α and IL‐6 secretion levels in aortic ECs. Each group included *n* = 6 animals. **p < 0.01, ***p < 0.001, ****p < 0.0001 between groups.

The development of AS is closely associated with vascular endothelial dysfunction, mitochondrial homeostasis disruption, and inflammatory responses. To investigate the role of FOXM1 in the mechanism by which PM@RSV NPs affect AS progression, an ApoE^−/−^ mouse model of AS induced by an HFD was used to evaluate the effects of PM@RSV NPs on AS lesions and their underlying molecular mechanisms (Figure 6A).

H&E staining showed that the AS model group exhibited significant intimal thickening and well‐defined plaque formation in the aorta, indicating vascular remodeling and the development of atherosclerotic lesions. Following PM@RSV NPs treatment, intimal thickening was notably alleviated and plaque boundaries appeared blurred. The RSV NPs and sh‐NC groups also showed varying degrees of improvement. In contrast, FOXM1 knockdown resulted in the reappearance of clear plaque structures and increased intimal thickness. Oil Red O staining showed that, relative to the AS model group, both RSV NPs and sh‐NC groups exhibited a reduction in lipid accumulation within aortic plaques, with the PM@RSV NPs group displaying the smallest red‐stained area and the lowest lipid content. Conversely, FOXM1 knockdown led to a marked increase in lipid deposition. Masson's trichrome staining demonstrated that the PM@RSV NPs group had relatively smaller plaque areas and a lower proportion of collagen‐positive regions, while the RSV NPs and sh‐NC groups showed intermediate levels. In the FOXM1 knockdown group, blue‐stained collagen areas were noticeably increased, indicating elevated collagen content (Figure 6B–C). These results suggest that PM@RSV NPs may modulate plaque size, lipid deposition, and fibrotic remodeling through activation of the FOXM1 pathway.

Mitochondrial oxidative stress in flow cytometry‐sorted ECs was assessed using the MitoSOX probe. The results showed that mitochondrial ROS levels were significantly reduced in the PM@RSV NPs group compared with the AS group, indicating that PM@RSV NPs effectively alleviated AS‐associated oxidative stress. However, this effect was markedly reversed upon FOXM1 knockdown, with ROS levels significantly increasing again (Figure 6D,E). TEM of ECs further confirmed these findings. In the AS group, EC mitochondria exhibited disrupted cristae and pronounced swelling. In contrast, mitochondria in the PM@RSV NPs group displayed restored morphology, while FOXM1 knockdown led to renewed mitochondrial damage (Figure 6F). ATP production assays in ECs showed that the PM@RSV NPs group had significantly increased ATP levels, whereas ATP production was markedly reduced following FOXM1 knockdown (Figure 6G), further confirming that PM@RSV NPs promote mitochondrial homeostasis via FOXM1, thereby enhancing endothelial energy metabolism. Given the pivotal role of mitochondrial dysfunction in AS progression, these findings suggest that PM@RSV NPs may improve mitochondrial energy supply by activating FOXM1, enhancing EC survival and functional recovery.

WB analysis of mitochondrial dynamics‐related proteins in isolated ECs revealed that PM@RSV NPs treatment significantly upregulated the expression of mitochondrial fusion proteins OPA1 and MFN2, while downregulating the expression of mitochondrial fission proteins DRP1 and FIS1. These changes were markedly reversed upon FOXM1 knockdown (Figure 6H,I). Imbalances in mitochondrial dynamics often lead to disrupted energy metabolism and impaired cellular function. These results suggest that PM@RSV NPs may promote mitochondrial fusion and suppress excessive mitochondrial fission via FOXM1, maintaining mitochondrial homeostasis and ensuring the adaptive survival and functional restoration of vascular ECs in the AS microenvironment.

Chronic inflammation represents a key driving mechanism in AS progression. WB analysis of isolated ECs showed that PM@RSV NPs markedly decreased the expression of the vascular inflammatory adhesion molecules ICAM‐1 and VCAM‐1 compared with the AS group, whereas FOXM1 knockdown substantially attenuated this inhibitory effect (Figure 6J,K). The high expression of vascular endothelial adhesion molecules promotes monocyte recruitment to lesion sites, exacerbating local inflammatory responses. PM@RSV NPs may suppress EC inflammation via FOXM1, thereby reducing monocyte adhesion and infiltration. In addition, ELISA analysis of ECs further demonstrated that PM@RSV NPs significantly reduced the secretion of pro‐inflammatory cytokines TNF‐α and IL‐6, while these effects were significantly reversed after FOXM1 knockdown (Figure 6L,M). Inflammation initiates and accelerates AS lesion progression. PM@RSV NPs inhibited the inflammatory cascade through FOXM1 activation, thereby reducing the inflammatory burden in the AS microenvironment. This mechanism may provide a new therapeutic strategy for anti‐AS treatment.

In vivo experiments demonstrated that PM@RSV NPs effectively improved the AS microenvironment by reducing plaque burden, enhancing plaque stability, improving mitochondrial function, promoting energy metabolism, and suppressing inflammatory responses. These results highlight the potent anti‐AS potential of PM@RSV NPs and underscore the central role of FOXM1 as a key therapeutic target in ameliorating AS pathology.

### 3.9 PM@RSV NPs Activate FOXM1 to Alleviate EC Senescence and Promote Vascular Regeneration

**FIGURE 7:**
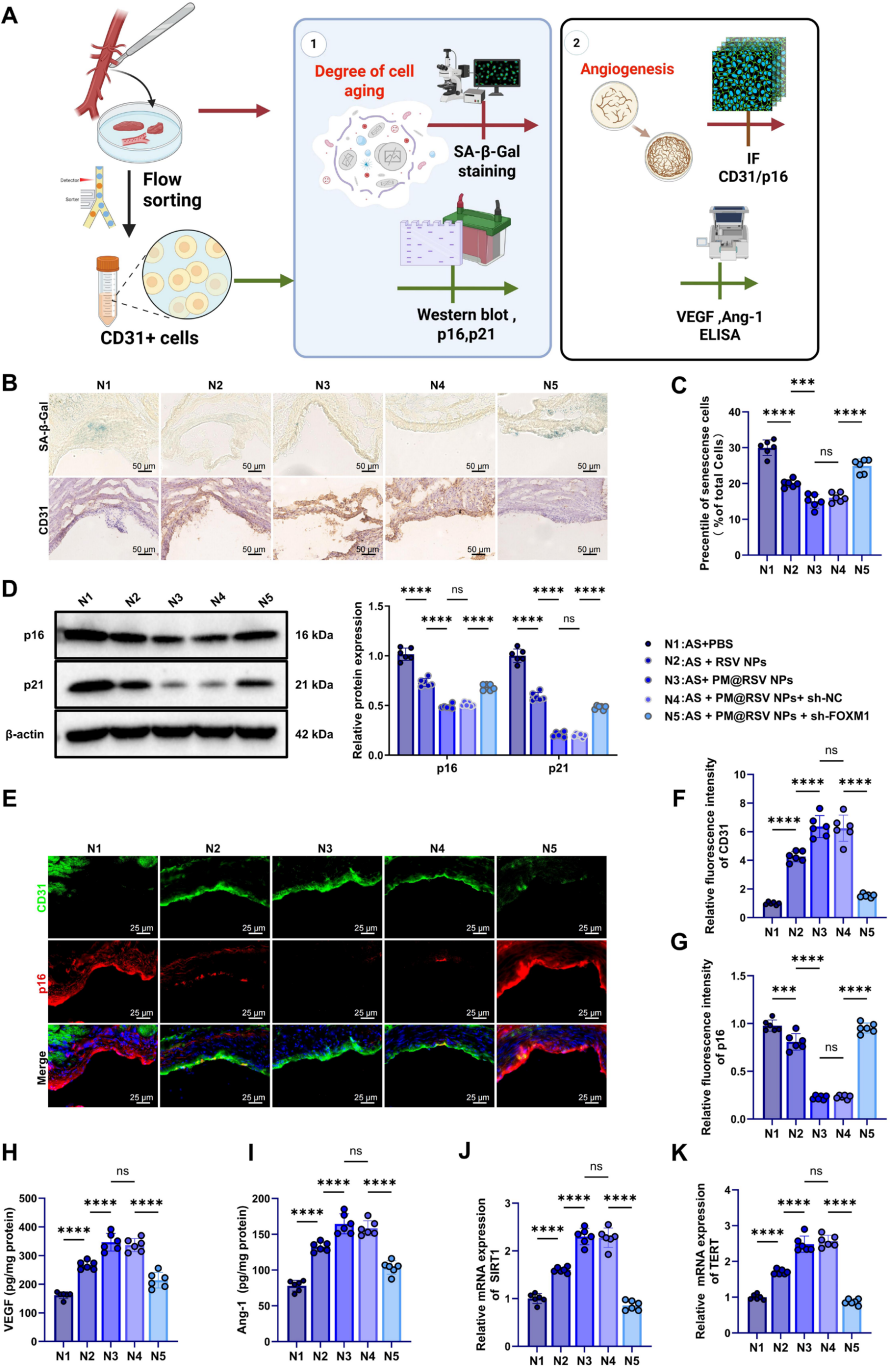
PM@RSV NPs promoted vascular endothelial proliferation, inhibited senescence, and improved the AS microenvironment via FOXM1 activation. (A) Schematic diagram of the experimental procedure (Created in BioRender); (B, C) SA‐β‐Gal staining to detect the proportion of senescent cells in aortic tissues (endothelial localization determined using adjacent sections stained for CD31 by IHC), bar = 50 μm; (D) Western blot analysis of p16 and p21 protein expression in aortic CD31^+^ ECs; (E, G) Immunofluorescence detection of CD31 and p16 expression in aortic tissues and corresponding quantification, bar = 25 μm; (H, I) ELISA detection of VEGF and Ang‐1 secretion levels in aortic CD31^+^ ECs; (J, K) RT‐qPCR analysis of SIRT1 and TERT mRNA expression levels in aortic CD31^+^ ECs. Each group included *n* = 6 animals. ***p < 0.001, ****p < 0.0001 between groups.

EC senescence and impaired vascular regeneration are key pathological mechanisms underlying the development of AS. To further elucidate the mechanism by which PM@RSV NPs improve endothelial function in vivo, an ApoE^−/−^ mouse model of AS induced by an HFD was used to systematically evaluate the effects of PM@RSV NPs on endothelial senescence, DNA repair, and vascular regeneration (Figure 7A).

SA‐β‐Gal staining combined with CD31 immunohistochemical co‐localization showed that, compared with the AS group, the RSV‐NPs group significantly reduced the proportion of SA‐β‐Gal‐positive cells, while the PM@RSV NPs group further decreased the accumulation of senescent cells. In contrast, FOXM1 knockdown led to a rebound in the proportion of SA‐β‐Gal‐positive cells, suggesting that FOXM1 may be a key regulatory factor mediating the anti‐senescence effects of PM@RSV NPs (Figure 7B,C). Furthermore, WB analysis of the senescence‐related proteins p16 and p21 in CD31^+^ ECs revealed that the PM@RSV NPs treatment group significantly downregulated their expression, whereas this inhibitory effect was markedly reversed after FOXM1 knockdown (Figure 7D).

Impaired vascular regeneration is another essential feature of AS progression. Immunofluorescence staining was performed to evaluate the co‐expression of the angiogenesis marker CD31 and the senescence marker p16 in aortic tissues. The results showed that CD31 expression (green) was enhanced after PM@RSV NPs treatment, indicating increased neovascularization, while FOXM1 knockdown led to a reduction in CD31 expression (Figure 7E,F). Consistent with the WB results, the expression of p16 further confirmed that PM@RSV NPs alleviated EC senescence through FOXM1 activation (Figure 7E,G).

Furthermore, ELISA results of CD31^+^ ECs lysates demonstrated that PM@RSV NPs significantly upregulated the expression of key angiogenic factors VEGF and Ang‐1, while the expression levels of these factors markedly decreased following FOXM1 knockdown (Figure 7H,I). These results suggest that PM@RSV NPs may enhance angiogenic capacity by activating the FOXM1‐mediated VEGF/Ang‐1 signaling pathway, promoting arterial repair and improving the AS microenvironment. Enhanced angiogenesis not only contributes to the restoration of endothelial integrity but may also improve local tissue perfusion, thus reducing the risk of ischemic injury.

To further examine whether PM@RSV NPs preserve EC energy metabolism and suppress senescence, SIRT1 and TERT expression was measured in CD31^+^ ECs isolated from aortic tissues. RT‐qPCR showed that PM@RSV NP treatment significantly increased SIRT1 and TERT expression compared with the AS group. However, FOXM1 knockdown markedly reduced their expression. These results suggest that PM@RSV NPs may enhance mitochondrial homeostasis, sustain energy metabolism, and delay EC senescence by upregulating SIRT1 and TERT through FOXM1 (Chen et al. 2014; Cui et al. 2023). SIRT1 plays a key role in regulating energy metabolism, oxidative stress response, and DNA repair, while TERT is closely associated with telomere maintenance and lifespan extension (Ji et al. 2022; Marchal 2024). These findings indicate that PM@RSV NPs may activate the SIRT1/TERT signaling axis through FOXM1, thereby enhancing the anti‐senescence capacity of ECs (Figure 7J,K).

In summary, PM@RSV NPs alleviated EC senescence and effectively promoted angiogenesis and vascular repair by activating the transcription factor FOXM1. These multi‐level improvements in endothelial function contributed to the mitigation of AS‐associated dysfunction and provided a novel strategy and theoretical basis for the precise regulation of the vascular microenvironment and targeted intervention in AS.

## 4 Discussion

During the progression of AS, EC senescence and mitochondrial dysfunction are key factors contributing to impaired vascular regeneration and the continuous deterioration of AS plaques (Pang et al. 2024; Zheng et al. 2024). Previous studies have shown that RSV possesses antioxidant and anti‐senescence properties (Parsamanesh et al. 2021; Li et al. 2019); however, its low bioavailability in vivo limits its clinical application (de Vries et al. 2021). In this study, PM@RSV NPs were synthesized to significantly enhance RSV accumulation and release efficiency at lesion sites, exhibiting excellent targeting capability and therapeutic potential. Unlike conventional non‐targeted drug delivery systems, the PM offers natural endothelial adhesion ability, facilitating precise drug delivery to atherosclerotic plaques while minimizing systemic toxicity.

This study identified FOXM1 as a critical regulator mediating the anti‐senescence and vascular repair effects of PM@RSV NPs. FOXM1 is a key transcription factor involved in the regulation of the cell cycle, DNA repair, and angiogenesis (Koo et al. 2012). Although widely studied in cancer, its role in AS remains poorly understood. PM@RSV NPs significantly upregulated FOXM1 expression and activated its downstream pathways, enhancing EC proliferation and repair. For the first time, we linked FOXM1 to mitochondrial functional restoration and the regulation of endothelial senescence, expanding the current understanding of its role in cardiovascular diseases.

Mitochondrial dysfunction is considered a major inducer of cellular senescence (Zheng et al. 2024; Headley et al. 2024; Zhang et al. 2024). JC‐1 staining, MitoSOX analysis, and ATP assays in this study confirmed that PM@RSV NPs effectively restored MMP, reduced ROS levels, and enhanced ATP production. Unlike conventional antioxidants that primarily scavenge free radicals, our strategy targeted the upstream regulator FOXM1 to achieve deeper mitochondrial functional restoration. This mechanism helped delay the onset of EC senescence, enhanced cellular functional stability and created favorable conditions for subsequent vascular repair.

Multi‐omics analysis represented a major highlight of this study. Using RNA‐seq in combination with GSEA, KEGG pathway enrichment, and LASSO regression analysis, we identified several key regulatory genes, including FOXM1, CDK1, PARP1, and IL1B, and constructed a PPI network, revealing their close association with cell cycle progression and immune regulation. Meanwhile, immune infiltration analysis demonstrated that PM@RSV NPs improved the local immune microenvironment and suppressed inflammatory responses, fostering favorable vascular regeneration conditions. Unlike previous studies that primarily focused on a single signaling pathway, this study adopted a multidimensional data integration approach, enabling a more comprehensive elucidation of the drug's mechanism of action.

In animal experiments, PM@RSV NPs significantly reduced plaque area and increased fibrous cap thickness. They also decreased the proportion of SA‐β‐Gal‐positive cells and the expression levels of p16 and p21, indicating a strong anti‐senescence effect. At the same time, ELISA confirmed that PM@RSV NPs upregulated angiogenic factors such as VEGF, thereby promoting neovascularization. Compared with conventional strategies focused solely on anti‐inflammation or pro‐angiogenesis, this study achieved a synergistic therapeutic effect by integrating anti‐senescence, anti‐inflammatory, and regenerative functions.

In vivo toxicity and biodistribution studies further confirmed the nanosystem's biosafety and targeting profile. The targeted cells/tissues evaluated in vivo were ECs within lesion‐associated vascular endothelium in ApoE^−/−^ mice, and biodistribution was assessed using DiR‐labeled PM@RSV NPs and CD31 co‐localization in aortic root plaques. PM@RSV NPs were primarily enriched in lesion‐associated vasculature, with no significant toxic responses observed in major organs based on H&E staining of the heart, liver, spleen, lung, kidney, and pancreas, and no detectable hepatic or renal toxicity based on serum ALT, AST, BUN, and Cr levels, supporting their translational potential. PM coating offers superior advantages in enhancing targeting ability and biocompatibility compared with polymeric or liposomal systems.

The PM@RSV NPs developed in this study exhibit multiple clinical potentials. First, the PM coating enabled precise targeting of AS lesions, significantly enhancing drug accumulation at lesion sites while minimizing off‐target effects on healthy tissues. This approach overcame the low bioavailability of RSV and demonstrated favorable therapeutic selectivity. Second, by activating the FOXM1, the nanosystem exerted synergistic effects in improving mitochondrial function, delaying endothelial senescence, and promoting vascular regeneration. These effects contributed to a systemic alleviation of AS progression, surpassing the limitations of conventional therapies that primarily focus on lipid‐lowering or anti‐inflammatory mechanisms. Third, the study demonstrated multi‐parametric therapeutic benefits in the ApoE^−/−^ mouse model, including reduced plaque area, increased fibrous cap thickness, enhanced angiogenesis, and decreased inflammation. These findings underscore the system's multi‐target and integrated regulatory characteristics, aligning with the therapeutic needs of cardiovascular diseases characterized by complex, multi‐mechanism comorbidities. Therefore, PM@RSV NPs offer a novel strategy for early intervention and may also serve as an effective complement to conventional therapies, providing a safer and more precise personalized treatment option for high‐risk AS patients.

Despite the promising findings in mechanistic investigation and therapeutic validation, this study has several noteworthy limitations. First, although the mouse model successfully mimicked the basic features of human AS, differences in vascular architecture and immune responses remain, necessitating further validation in large animal models or preclinical humanized models. Second, although FOXM1 was identified as a key regulatory factor, its upstream regulatory mechanisms remain unclear, and the involvement of other signaling pathways in the effects of RSV cannot be entirely excluded. Third, the study primarily focused on short‐term intervention. It did not evaluate the potential long‐term effects of PM@RSV NPs on the immune system, chronic toxicity, or possible tissue accumulation, all of which are critical for clinical translation. Moreover, challenges remain in achieving large‐scale, standardized production and stable preservation of the drug delivery system. Therefore, future studies should focus on mechanistic refinement, comprehensive toxicological evaluation, and process optimization to ensure the feasibility and safety of clinical application.

Based on the current findings, PM@RSV NPs demonstrated significant advantages in treating AS. Future research directions may proceed along several avenues. First, regarding mechanistic studies, it is necessary to further elucidate the upstream regulatory network of FOXM1 and its interactions with other mitochondrial regulators, such as PGC‐1α and SIRT1, to identify potential synergistic targets. Second, in terms of application development, exploring the combined use of this nanosystem with current clinical standard therapies, such as statins and PCSK9 inhibitors, is recommended to investigate synergistic effects and dose optimization strategies. Third, the therapeutic scope may include additional indications, such as vascular complications associated with diabetes or post‐ischemic repair in cardiovascular and cerebrovascular events. Moreover, before clinical translation, it is essential to advance key steps, including process scale‐up, GMP‐grade manufacturing, stability assessment, and toxicological evaluation in large animal models. The ultimate goal is to develop a personalized nanomedicine platform based on PM@RSV NPs, enabling a strategic transition from merely “delaying lesion progression” to actively “repairing vascular damage,” thereby offering a novel and comprehensive therapeutic approach for the management of chronic vascular diseases.

In this study, we developed PM@RSV NP that effectively enhanced mitochondrial bioenergetics in ECs, attenuated cellular senescence, and stimulated vascular regeneration through FOXM1‐dependent mechanisms. Both in vitro and in vivo results demonstrated that PM@RSV NPs regulated DNA repair and mitochondrial metabolic pathways, reduced oxidative stress and inflammation, and enhanced endothelial function, thereby effectively delaying the progression of AS lesions. Preferential uptake by injured MAECs and lesion‐associated vascular endothelium, together with the absence of obvious cytotoxicity, major‐organ histological injury, or abnormal liver/kidney biochemical indices, further supported the targeting profile, biosafety, and clinical application potential of this nanosystem as a novel therapeutic strategy for treating AS.

This study revealed the critical role of FOXM1 in regulating mitochondrial function and DNA repair, and applied this mechanism to nanomedicine development, thereby enriching the molecular understanding of AS. Through PM modification, PM@RSV NPs achieved targeted delivery and enhanced the bioavailability and therapeutic efficacy of RSV. Its combined antioxidant, anti‐inflammatory, and pro‐angiogenic effects offer a new approach to AS therapy and hold significant potential for clinical translation.

## Author Contributions

**Li Xiao:** writing – original draft, project administration, methodology, investigation, formal analysis, data curation, conceptualization. **Zexin Zhan:** investigation, formal analysis, data curation, writing – review and editing. **Ping Liu:** supervision, validation, visualization, writing – review and editing. **Bing Qin:** writing – review and editing, writing – original draft, funding acquisition, supervision, methodology, conceptualization.

## Funding

This study was supported by the National Natural Science Foundation of China (No. 81701167) and the Natural Science Foundation of Guangdong Province, China (2017A030310360).

## Ethics Statement

All animal experiments were approved by the Animal Ethics Committee of Sun Yat‐Sen University (No. L102012018040Z).

## Conflicts of Interest

The authors declare no conflicts of interest.

## Supporting information


**Figure S1:** Assessment of EC senescence, mitochondrial dysfunction, and vascular repair/regeneration in the ApoE^−/−^ mouse model of AS. (A) Schematic diagram of AS model construction and experimental procedures in ApoE^−/−^ mice; (B) H&E staining, Oil Red O staining, and Masson's trichrome staining of the aortic sinus sections, bar = 200 μm; (C) Quantification of plaque formation and lipid deposition in aortic tissues; (D) Western blot analysis of senescence markers p16 and p21 in aortic CD31^+^ ECs; (E) Dual immunofluorescence staining to detect senescent ECs in aortic tissues, bar = 25 μm; (F) Western blot analysis and quantification of mitochondrial fusion proteins (OPA1, MFN2) and fission proteins (DRP1, FIS1) in aortic CD31^+^ ECs; (G‐H) Flow cytometric analysis of mitochondrial ROS levels in aortic CD31^+^ ECs using the MitoSOX fluorescent probe staining; (I) ATP assay to evaluate mitochondrial energy metabolism in aortic CD31^+^ ECs; (J) Immunofluorescence detection of angiogenic markers CD31 and VEGF in aortic tissues, bar = 25 μm; (K) Levels of SOD, CAT, and MDA in CD31^+^ ECs from aortic tissues; (L) ELISA analysis of pro‐inflammatory cytokines TNF‐α and IL‐6 in aortic CD31^+^ ECs. Six animals were used per group. Data are presented as mean ± standard deviation. ***p < 0.001, ****p < 0.0001 between groups.

**Figure S2:** Evaluation of the physical stability of PM@RSV NPs under different storage conditions. (A) Schematic diagram of the experimental workflow for the stability assessment of PM@RSV NPs; (B‐C) DLS analysis of particle size distribution and Zeta potential after 24 h of storage at 37°C; (D‐E) DLS analysis of particle size distribution and Zeta potential after 14 days of storage at 4°C. Experiments were performed in triplicate.

**Figure S3:** The PM@RSV NPs exhibited low cytotoxicity. (A) Schematic diagram of the biosafety evaluation workflow for MAECs treated with PM@RSV NPs; (B) Cell viability measured by CCK‐8 assay; (C, D) Cell viability assessed by Calcein‐AM/PI double staining, bar = 25 μm; (E, F) Apoptosis rate measured by flow cytometry. Cell experiments were performed in triplicate. ns indicates no significant difference between groups.

**Figure S4:** Cellular uptake analysis of PM@RSV NPs in ECs. (A) Schematic diagram of the experimental workflow for cellular uptake analysis of PM@RSV NPs in MAECs; (B) Quantification of PM@RSV NPs and RSV NPs uptake at 1 h and 4 h by flow cytometry; (C) Colocalization of Cy5‐labeled PM@RSV NPs with the lysosomal marker LAMP1 observed by confocal microscopy, bar = 25 μm; (D) Quantification of PM@RSV NPs uptake in normal MAECs and ox‐LDL‐induced injured MAECs by flow cytometry; (E) Flow cytometry analysis of PM@RSV NPs uptake after pretreatment with neutralizing antibodies against GPV and P‐selectin. Cell experiments were performed in triplicate. **p < 0.01, ****p < 0.0001 versus control.

**Figure S5:** Immune cell infiltration analysis. (A) Composition of 22 immune cell subtypes in arterial samples from control and treatment groups; (B) Boxplots showing differences in immune cell proportions between control and treatment groups; (C) FOXM1 expression was significantly negatively correlated with naïve B cells, resting dendritic cells, and resting NK cells. Control: *n* = 3; Treatment: *n* = 3.

**Figure S6:** Detection of FOXM1 expression. (A, B) Western blot analysis of FOXM1 protein knockdown efficiency by sh‐FOXM1‐1 and sh‐FOXM1‐2 in ox‐LDL‐treated ECs; (C) RT‐qPCR analysis of FOXM1 mRNA knockdown efficiency by sh‐FOXM1‐1 and sh‐FOXM1‐2 in ox‐LDL‐treated ECs; (D‐E) Western blot analysis of FOXM1 protein expression in ECs across different treatment groups; (F) RT‐qPCR analysis of FOXM1 mRNA expression in ECs across different treatment groups. Experiments were performed in triplicate. *p < 0.05, ***p < 0.001, ****p < 0.0001 versus control.

**Figure S7:** PM@RSV NPs exhibited favorable in vivo biosafety. (A) Schematic diagram of the experimental design and detection methods in the ApoE^−/−^ mouse model (Created in BioRender); (B) H&E staining of major organs (heart, liver, spleen, lung, kidney) to assess histological structure, bar = 100 μm; (C) Blood biochemical analysis of liver and kidney function indicators, including ALT, AST, BUN, and Cr levels. Each group included *n* = 6 animals. ns indicates no significant difference between groups.

**Figure S8:** PM@RSV NPs exhibited favorable in vivo targeting capability. (A) Schematic diagram of the experimental workflow showing DiR‐labeled PM@RSV NPs distribution in ApoE^−/−^ mice (Created in BioRender); (B) Representative ex vivo fluorescence imaging of major organs showing biodistribution of PM@RSV NPs; (C, D) Fluorescence signal and quantification in the aorta 24 h post‐injection; (E) CLSM image of PM@RSV NPs accumulation in AS plaques of aortic root sections, bar = 50 μm. Each group included *n* = 6 animals. ****p < 0.0001.

**Table S1:** RT‐qPCR primer sequence table.
**Table S2:** Primary antibody product details.

## Data Availability

The data that support the findings of this study are available on request from the corresponding author. The data are not publicly available due to privacy or ethical restrictions.
