# Supplementary figures and images for: Mechanistic Study of Platelet Membrane‐Coated Resveratrol Nanosystem in Mitochondrial Dysfunction and Endothelial Senescence During Atherosclerotic Lesion Development via FOXM1 Activation
Source: Aging Cell. 2026 Jul 21;25(8):e70632. doi: 10.1111/acel.70632 (PMC13387738; doi:10.1111/acel.70632)

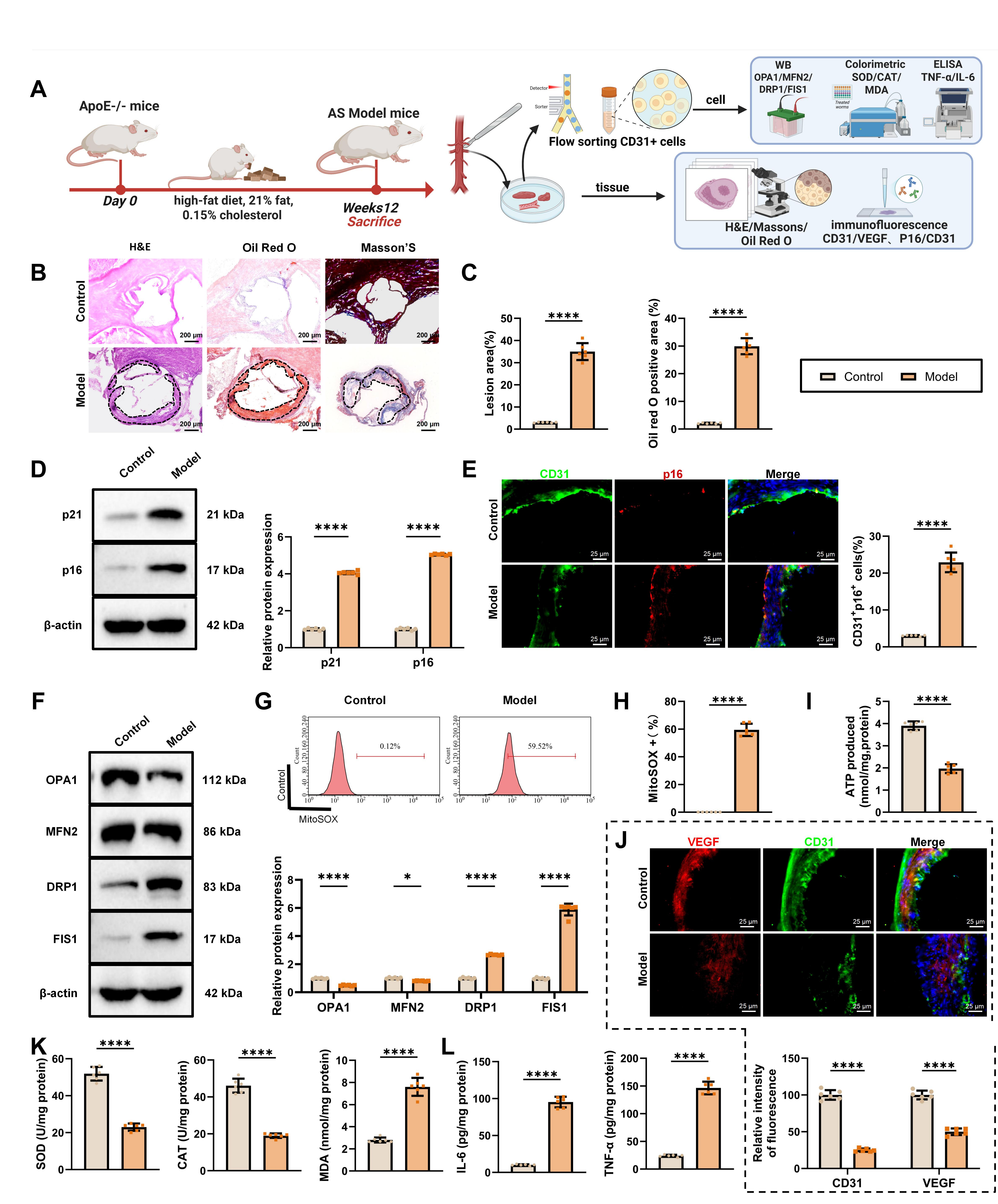

Supplement: Supplementary file 1 — Figure S1: Assessment of EC senescence, mitochondrial dysfunction, and vascular repair/regeneration in the ApoE−/− mouse model of AS. (A) Schematic diagram of AS model construction and experimental procedures in ApoE−/− mice; (B) H&E staining, Oil Red O staining, and Masson's trichrome staining of the aortic sinus sections, bar = 200 μm; (C) Quantification of plaque formation and lipid deposition in aortic tissues; (D) Western blot analysis of senescence markers p16 and p21 in aortic CD31+ ECs; (E) Dual immunofluorescence staining to detect senescent ECs in aortic tissues, bar = 25 μm; (F) Western blot analysis and quantification of mitochondrial fusion proteins (OPA1, MFN2) and fission proteins (DRP1, FIS1) in aortic CD31+ ECs; (G‐H) Flow cytometric analysis of mitochondrial ROS levels in aortic CD31+ ECs using the MitoSOX fluorescent probe staining; (I) ATP assay to evaluate mitochondrial energy metabolism in aortic CD31+ ECs; (J) Immunofluorescence detection of angiogenic markers CD31 and VEGF in aortic tissues, bar = 25 μm; (K) Levels of SOD, CAT, and MDA in CD31+ ECs from aortic tissues; (L) ELISA analysis of pro‐inflammatory cytokines TNF‐α and IL‐6 in aortic CD31+ ECs. Six animals were used per group. Data are presented as mean ± standard deviation. ***p < 0.001, ****p < 0.0001 between groups. [file ACEL-25-e70632-s005.png]

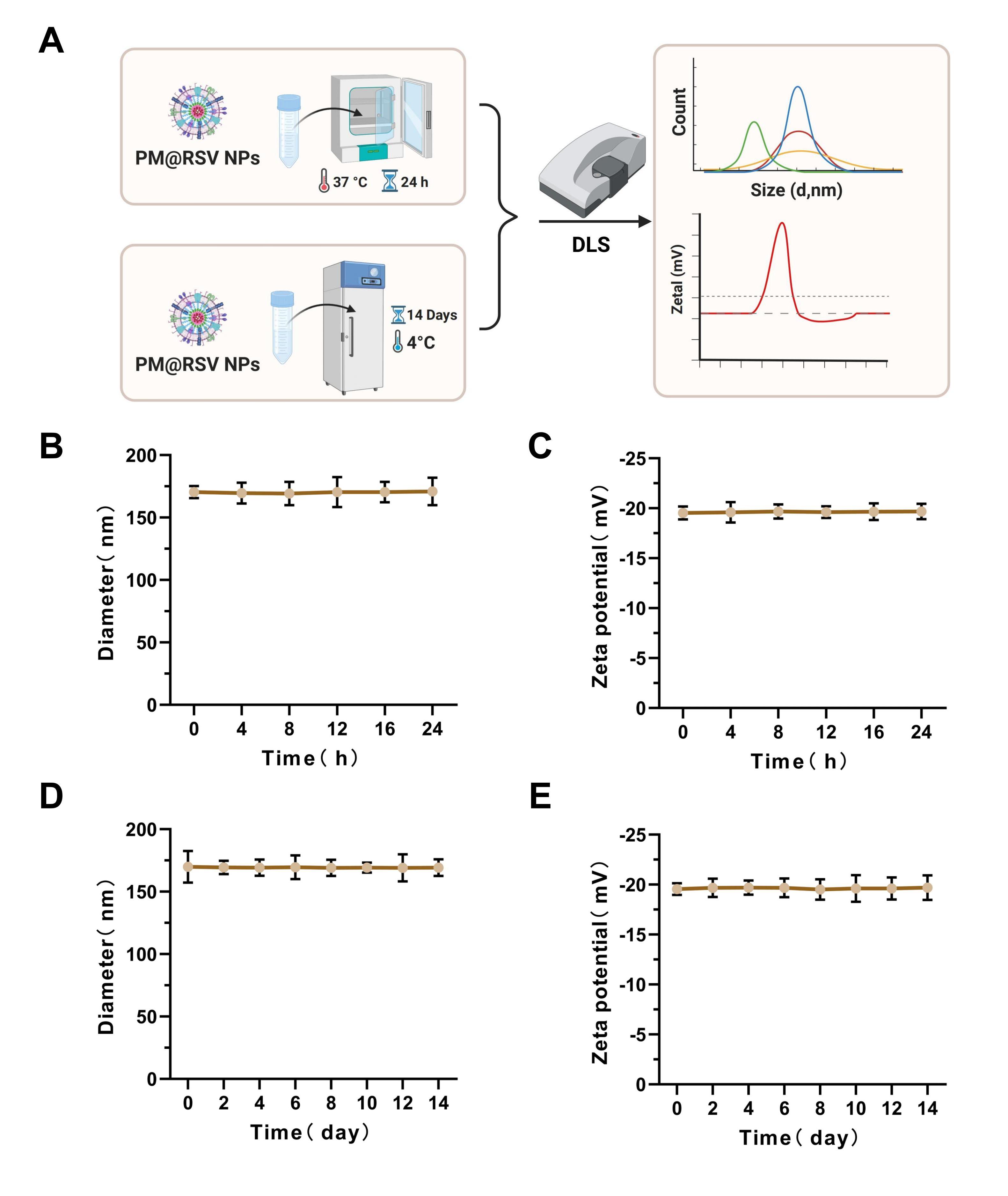

Supplement: Supplementary file 2 — Figure S2: Evaluation of the physical stability of PM@RSV NPs under different storage conditions. (A) Schematic diagram of the experimental workflow for the stability assessment of PM@RSV NPs; (B‐C) DLS analysis of particle size distribution and Zeta potential after 24 h of storage at 37°C; (D‐E) DLS analysis of particle size distribution and Zeta potential after 14 days of storage at 4°C. Experiments were performed in triplicate. [file ACEL-25-e70632-s009.jpg]

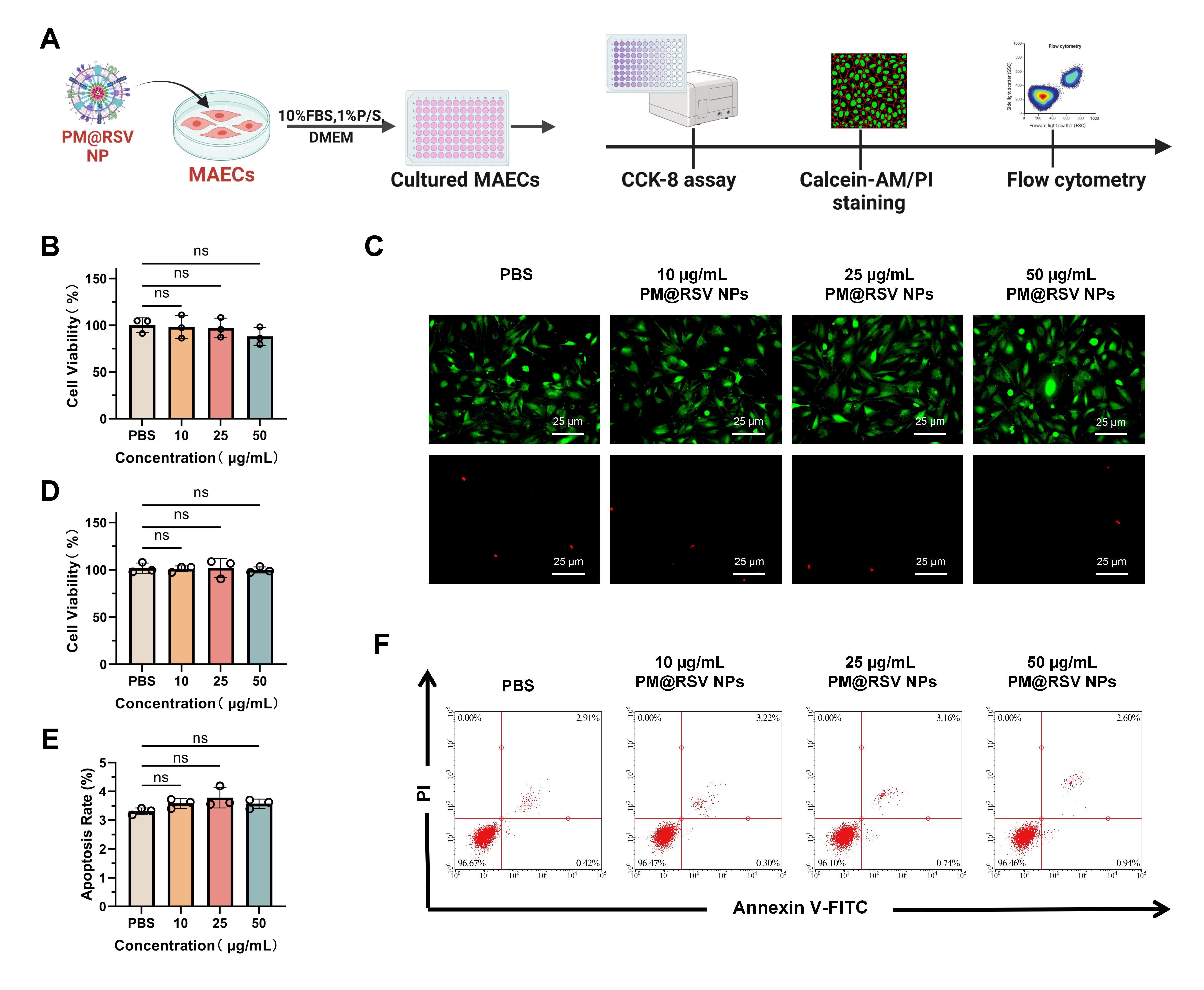

Supplement: Supplementary file 3 — Figure S3: The PM@RSV NPs exhibited low cytotoxicity. (A) Schematic diagram of the biosafety evaluation workflow for MAECs treated with PM@RSV NPs; (B) Cell viability measured by CCK‐8 assay; (C, D) Cell viability assessed by Calcein‐AM/PI double staining, bar = 25 μm; (E, F) Apoptosis rate measured by flow cytometry. Cell experiments were performed in triplicate. ns indicates no significant difference between groups. [file ACEL-25-e70632-s008.jpg]

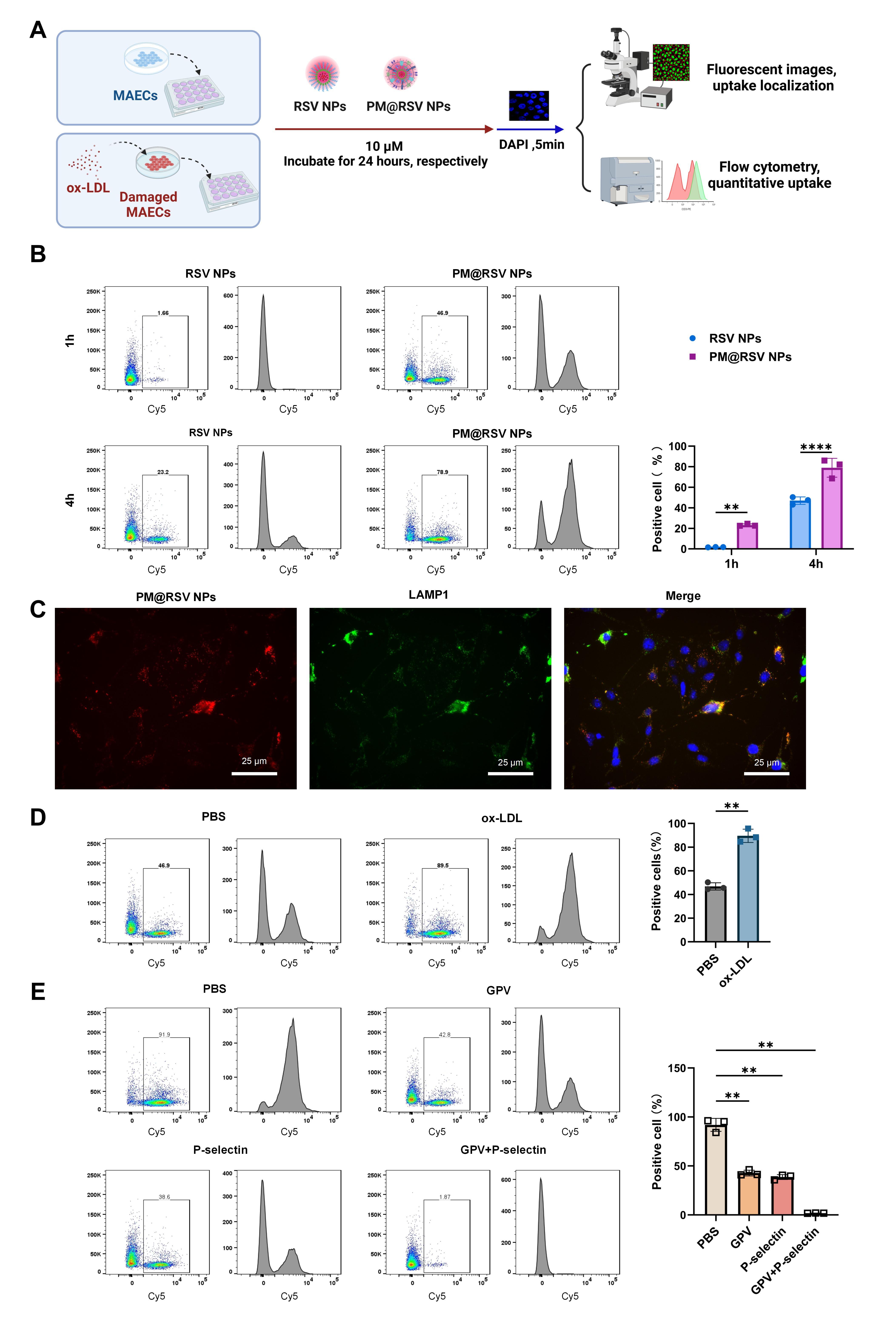

Supplement: Supplementary file 4 — Figure S4: Cellular uptake analysis of PM@RSV NPs in ECs. (A) Schematic diagram of the experimental workflow for cellular uptake analysis of PM@RSV NPs in MAECs; (B) Quantification of PM@RSV NPs and RSV NPs uptake at 1 h and 4 h by flow cytometry; (C) Colocalization of Cy5‐labeled PM@RSV NPs with the lysosomal marker LAMP1 observed by confocal microscopy, bar = 25 μm; (D) Quantification of PM@RSV NPs uptake in normal MAECs and ox‐LDL‐induced injured MAECs by flow cytometry; (E) Flow cytometry analysis of PM@RSV NPs uptake after pretreatment with neutralizing antibodies against GPV and P‐selectin. Cell experiments were performed in triplicate. **p < 0.01, ****p < 0.0001 versus control. [file ACEL-25-e70632-s002.jpg]

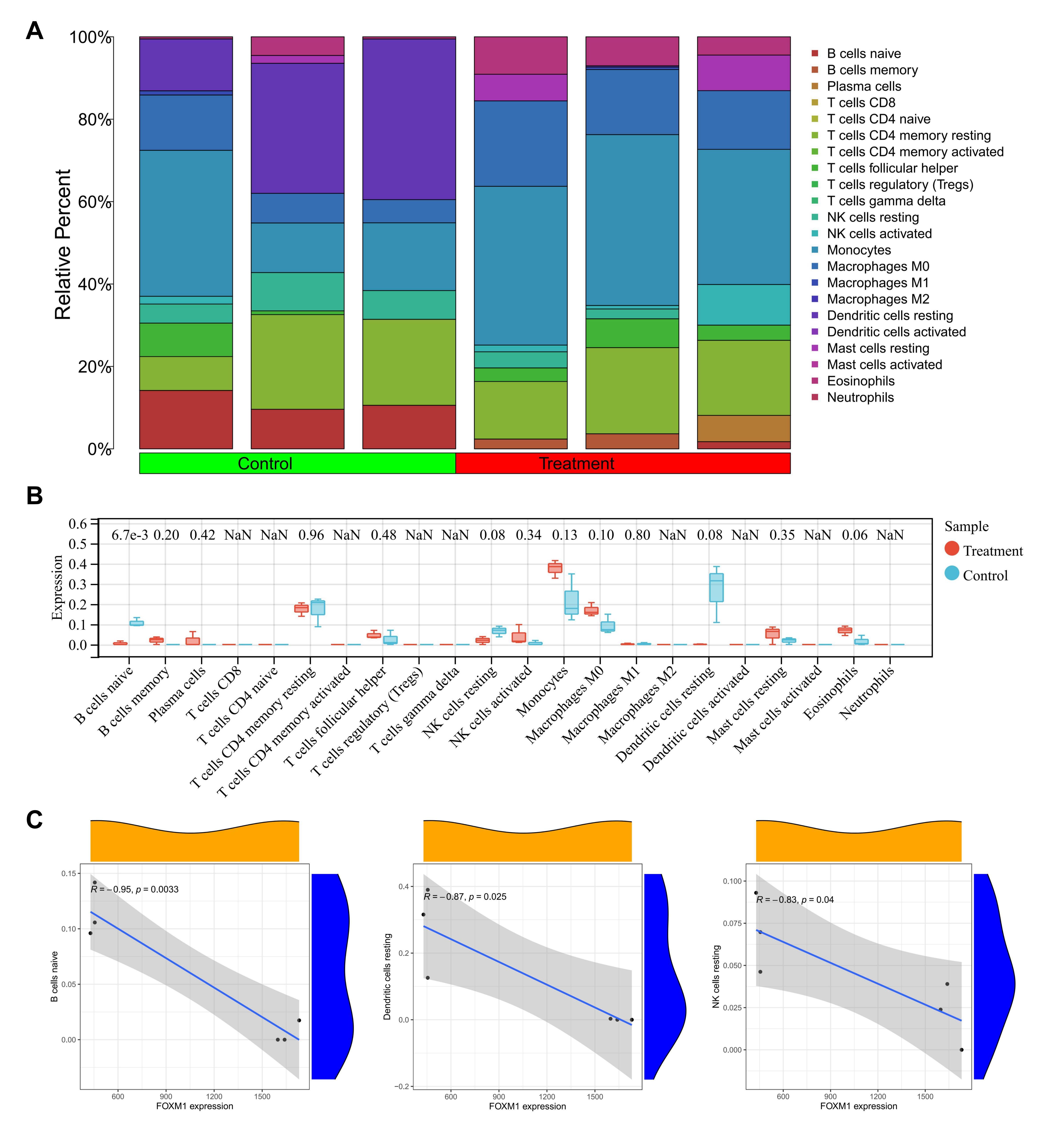

Supplement: Supplementary file 5 — Figure S5: Immune cell infiltration analysis. (A) Composition of 22 immune cell subtypes in arterial samples from control and treatment groups; (B) Boxplots showing differences in immune cell proportions between control and treatment groups; (C) FOXM1 expression was significantly negatively correlated with naïve B cells, resting dendritic cells, and resting NK cells. Control: n = 3; Treatment: n = 3. [file ACEL-25-e70632-s007.jpg]

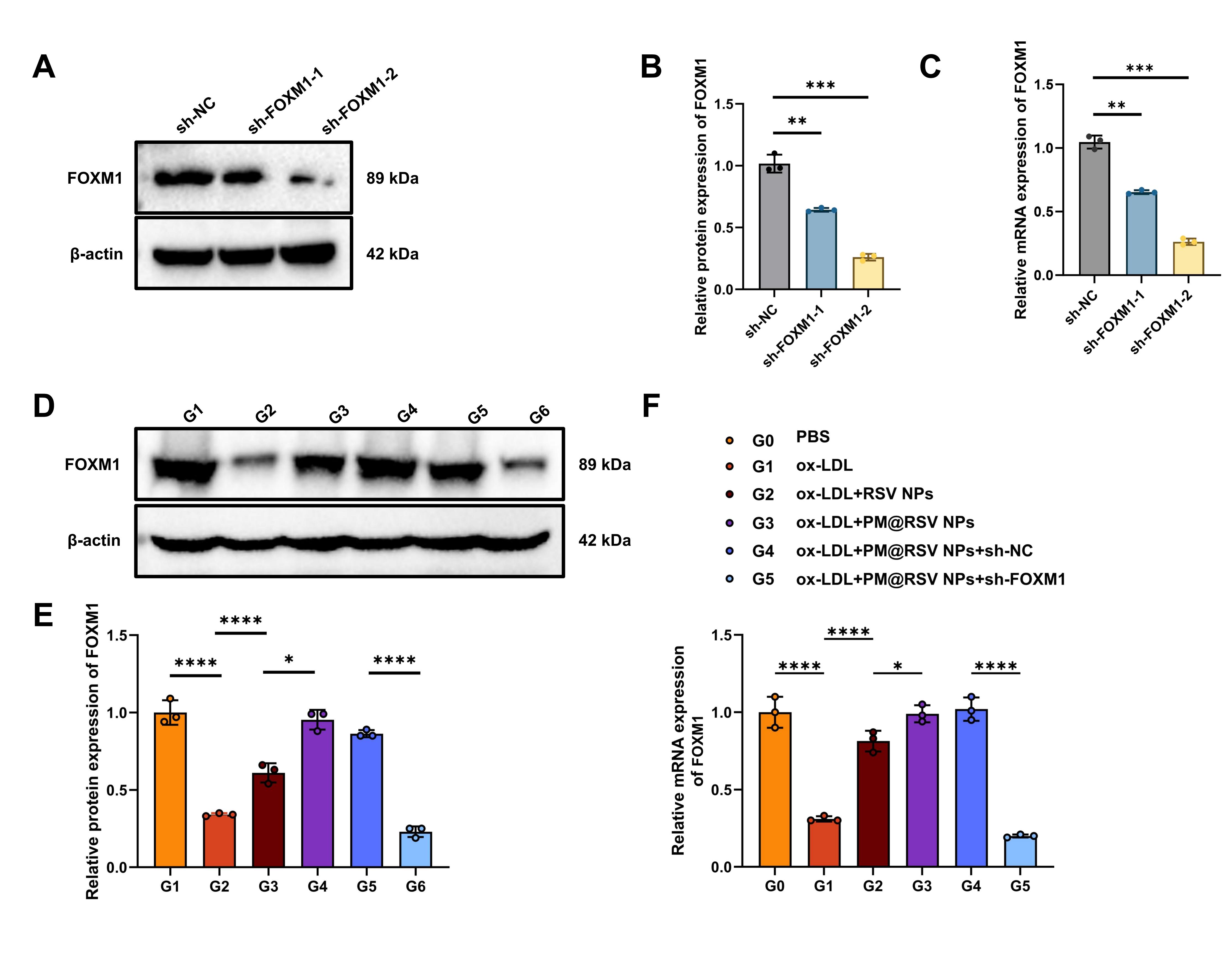

Supplement: Supplementary file 6 — Figure S6: Detection of FOXM1 expression. (A, B) Western blot analysis of FOXM1 protein knockdown efficiency by sh‐FOXM1‐1 and sh‐FOXM1‐2 in ox‐LDL‐treated ECs; (C) RT‐qPCR analysis of FOXM1 mRNA knockdown efficiency by sh‐FOXM1‐1 and sh‐FOXM1‐2 in ox‐LDL‐treated ECs; (D‐E) Western blot analysis of FOXM1 protein expression in ECs across different treatment groups; (F) RT‐qPCR analysis of FOXM1 mRNA expression in ECs across different treatment groups. Experiments were performed in triplicate. *p < 0.05, ***p < 0.001, ****p < 0.0001 versus control. [file ACEL-25-e70632-s006.jpg]

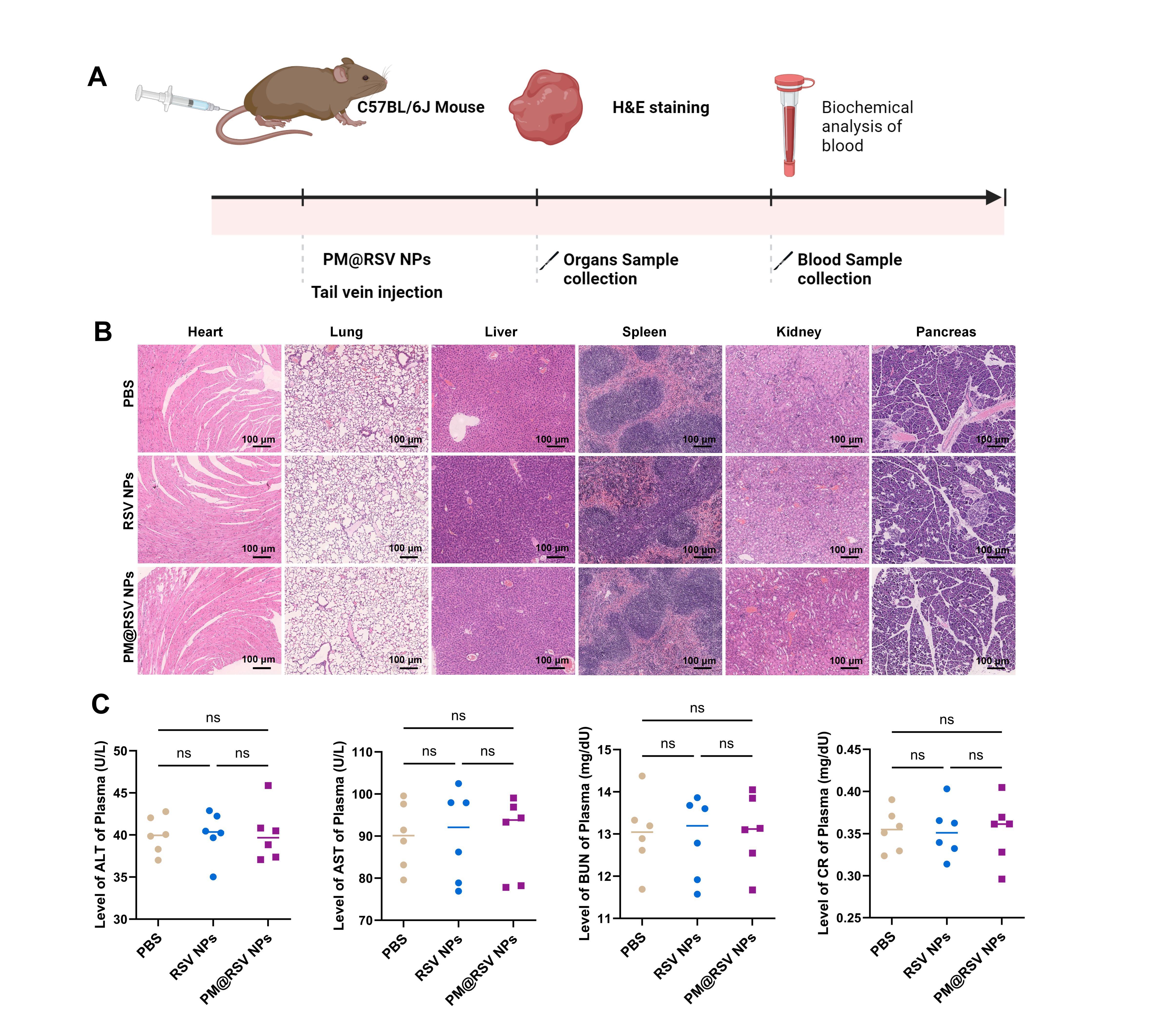

Supplement: Supplementary file 7 — Figure S7: PM@RSV NPs exhibited favorable in vivo biosafety. (A) Schematic diagram of the experimental design and detection methods in the ApoE−/− mouse model (Created in BioRender); (B) H&E staining of major organs (heart, liver, spleen, lung, kidney) to assess histological structure, bar = 100 μm; (C) Blood biochemical analysis of liver and kidney function indicators, including ALT, AST, BUN, and Cr levels. Each group included n = 6 animals. ns indicates no significant difference between groups. [file ACEL-25-e70632-s003.jpg]

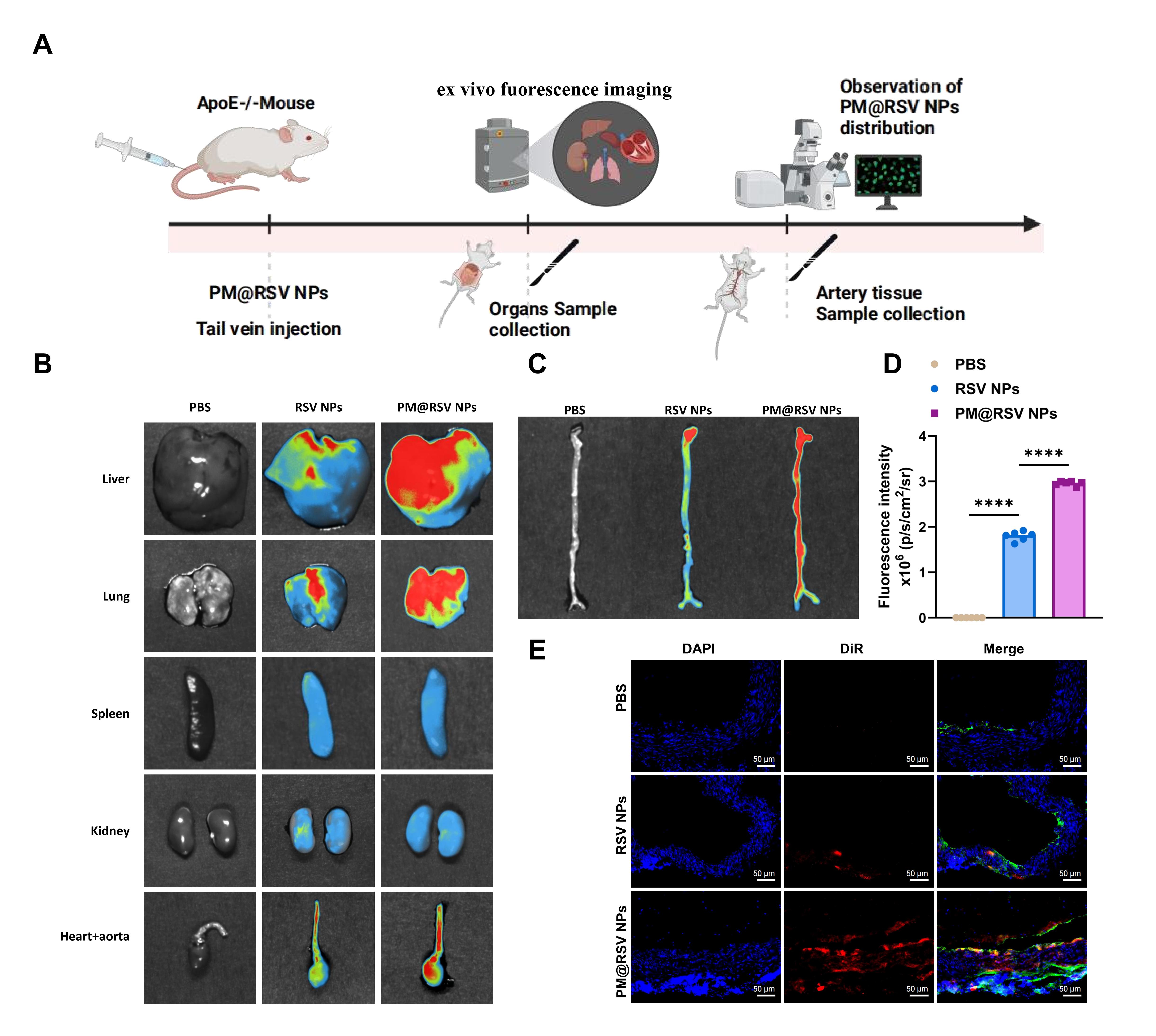

Supplement: Supplementary file 8 — Figure S8: PM@RSV NPs exhibited favorable in vivo targeting capability. (A) Schematic diagram of the experimental workflow showing DiR‐labeled PM@RSV NPs distribution in ApoE−/− mice (Created in BioRender); (B) Representative ex vivo fluorescence imaging of major organs showing biodistribution of PM@RSV NPs; (C, D) Fluorescence signal and quantification in the aorta 24 h post‐injection; (E) CLSM image of PM@RSV NPs accumulation in AS plaques of aortic root sections, bar = 50 μm. Each group included n = 6 animals. ****p < 0.0001. [file ACEL-25-e70632-s001.jpg]
