# Supplementary material for: Mechanistic Study of Platelet Membrane‐Coated Resveratrol Nanosystem in Mitochondrial Dysfunction and Endothelial Senescence During Atherosclerotic Lesion Development via FOXM1 Activation
Source: Aging Cell. 2026 Jul 21;25(8):e70632. doi: 10.1111/acel.70632 (PMC13387738; doi:10.1111/acel.70632)
Supplement: Supplementary file 9 — Table S1: RT‐qPCR primer sequence table. Table S2: Primary antibody product details. [file ACEL-25-e70632-s004.doc]

Table S1. RT-qPCR primer sequence table

| Gene Name | Primer Sequence |
| --- | --- |
| β-actin (mouse) | Forward: 5'-CACTGTCGAGTCGCGTCC-3' |
|  | Reverse: 5'-CGCAGCGATATCGTCATCCA-3' |
| FoxM1(mouse) | Forward: 5'-GAATGGCCAACATCCCGAAG-3' |
|  | Reverse: 5'-GTTGGGCCCCACTCTACCTT-3' |
| Pcna (mouse) | Forward: 5'-TGGTAGTTGTCGCTGTAGGC-3' |
|  | Reverse: 5'-ATCAGGCGTGCCTCAAACAT-3' |
| Ki-67 (mouse) | Forward: 5'-TCATTGACCGCTCCTTTAGGT-3' |
|  | Reverse: 5'-TTGACCTTCCCCATCAGGGT-3' |
| Sirt1 (mouse) | Forward: 5'-CGGCTACCGAGGTCCATATAC-3' |
|  | Reverse: 5'-ACAATCTGCCACAGCGTCAT-3' |
| Tert (mouse) | Forward:5'-CTGTGCCTACCAGGGGAGAT-3' |
|  | Reverse:5'-GGCCTTGAGCCCAGAAAGAT-3' |

**Table S2. Primary antibody product details**

| **Antibody Name** | **Catalog Number** | **Dilution Ratio** | **Manufacturer** | **Origin** |
| --- | --- | --- | --- | --- |
| FoxM1 | ab207298 | 1:1000 | Abcam | UK |
| OPA1 | ab42364 | 1:2000 | Abcam | UK |
| MFN2 | ab124773 | 1:1000 | Abcam | UK |
| DRP1 | ab184247 | 1:2000 | Abcam | UK |
| FIS1 | ab229969 | 1:2000 | Abcam | UK |
| p16 | ab51243 | 1:1000 | Abcam | UK |
| p21 | ab109199 | 1:2000 | Abcam | UK |
| Cyclin D1 | ab16663 | 1:1000 | Abcam | UK |
| Cyclin E | ab33911 | 1:1000 | Abcam | UK |
| CDK2 | ab32147 | 1:1000 | Abcam | UK |
| ICAM1 | ab222736 | 1:1000 | Abcam | UK |
| VCAM1 | ab134047 | 1:2000 | Abcam | UK |
| CD62P(P-Selectin) | ab255822 | 1:1000 | Abcam | UK |
| CD42d (GPV) | PA5-47889 | 1:1000 | ThermoFisher | USA |
| CD42a (GPIX) | MA5-50570 | 1:1000 | ThermoFisher | USA |
| CD41（αIIb） | PA5-79527 | 1:1000 | ThermoFisher | USA |
| CD61( GPIIIa,β3) | MA5-32077 | 1:1000 | ThermoFisher | USA |
| CD29（β1） | MA5-17103 | 1:1000 | ThermoFisher | USA |
| β-actin | ab6276 | 1:5000 | Abcam | UK |
